# Identifying and ranking non-traditional risk factors for cardiovascular disease prediction in people with type 2 diabetes

**DOI:** 10.1038/s43856-025-00785-y

**Published:** 2025-03-14

**Authors:** Katarzyna Dziopa, Nishi Chaturvedi, Folkert W. Asselbergs, Amand F. Schmidt

**Affiliations:** 1 Institute of Health Informatics, University College London, London, UK; 2 Institute of Cardiovascular Science, Faculty of Population Health Sciences, University College London, London, UK; 3 Department of Cardiology, Amsterdam Cardiovascular Science, Amsterdam University Medical Centers, University of Amsterdam, Amsterdam, The Netherlands; 4 Department of Population Science and Experimental Medicine, University College London, London, UK; 5 The National Institute for Health Research UCL Hospitals Biomedical Research Centre, University College London, London, UK; 6 UCL BHF Research Accelerator Centre, London, UK

**Keywords:** Cardiology, Disease prevention, Cardiovascular diseases, Metabolic disorders

## Abstract

**Background:**

Cardiovascular disease **(**CVD) prediction models perform poorly in people with type 2 diabetes (T2DM). We aimed to identify potentially non-traditional CVD predictors for six facets of CVD (including coronary heart disease, ischemic stroke, heart failure, and atrial fibrillation) in people with T2DM.

**Methods:**

We analysed data on 600+ features from the UK Biobank, stratified by history of CVD and T2DM: 459,142 participants without diabetes or CVD, 14,610 with diabetes but without CVD, and 4432 with diabetes and CVD. A penalised generalized linear model with a binomial distribution was used to identify CVD-related features. Subsequently, a 20% hold-out set was used to replicate identified features and provide an importance based ranking.

**Results:**

Here we show that non-traditional risk factors are of particular importance in people with diabetes. Classical CVD risk factors (e.g. family history, high blood pressure) rank highly in people without diabetes. For individuals with T2DM but no CVD, top predictors include cystatin C, self-reported health satisfaction, biochemical measures of ill health. In people with diabetes and CVD, key predictors are self-reported ill health and blood cell counts. Unique diabetes-related risk factors include dietary patterns, mental health and biochemistry measures (e.g. oestradiol, rheumatoid factor). Adding these features improves risk stratification; per 1000 people with diabetes, 133 CVD and 165 HF cases receive a higher risk.

**Conclusions:**

This study identifies numerous replicated non-traditional CVD risk factors for people with T2DM, providing insight to improve guideline recommended risk prediction models which currently overlook these features.

## Plain language summary

People living with type-2 diabetes have a higher chance of developing problems with their heart or blood circulation, known as cardiovascular disease. Cardiovascular disease is a major cause of illness and death in people with diabetes. Commonly used risk factors for cardiovascular disease are unable to identify who will develop disease in future. This study aims to uncover less well-known cardiovascular risk factors of particular importance to identify people with diabetes who are likely to develop heart disease in the next 10 years. Our findings reveal the importance of self-reported health issues, non-cholesterol blood test results, dietary patterns, and mental health in identifying people with diabetes who will develop cardiovascular disease. Overall, these factors can improve cardiovascular disease care and self-management in people with type-2 diabetes.

## Abstract (web summary)

Dziopa et al. identify and rank non-traditional risk factors for cardiovascular disease in people with type 2 diabetes. Findings reveal key predictors of future cardiovascular events such as self-reported ill health, non-lipid related blood biomarkers, dietary patterns, and mental health factors.

## Introduction

We, and others^1,2^, have shown that cardiovascular (CVD) risk prediction models do not perform well in people with type 2 diabetes (T2DM). Importantly, performance did not differ meaningfully between 22 CVD risk prediction models, with c-statistics, estimating discrimination, close to 0.70^3^, while for general population this is 0.88 in women and 0.86 in men (based on the QRISK3^4^). This near-constant lower performance of most CVD prediction rules in people with T2DM likely reflect the considerable overlap in considered predictor variables, such as age, sex, blood pressure, and cholesterol reflecting a focus on features with a proven CVD association. The relative poor performance in people with T2DM identifies a need to consider less classical features for CVD prediction.

The need to include nonstandard predictors for CVD has previously been shown by Wang et al. ^5^, where up to 20% of participants with coronary disease did not possess conventional CVD risk factors, and 40% presented with only a single risk factor. This is especially important because individuals with T2DM exhibit an elevated risk of cardiovascular morbidity and mortality and are disproportionately affected by CVD compared to people without T2DM^6,7^. The rising prevalence of T2DM, combined with advancements in post-CVD event care, contributes to a growing population at risk for CVD events^8,9^. Given that T2DM itself is a risk factor for CVD, a substantial number of people with diabetes are living with established CVD. This highlights the need to determine to what extent CVD risk factors in people with diabetes differ by history of CVD.

The UK Biobank (UKB) was initiated to further understanding of health in all its facets, and therefore collects measurements irrespective of clinical indication. For example, in clinical settings glucose and glycated haemoglobin (HbA_1c_) are typically only measured in people with, or at risk for, diabetes. In the UKB these features have been measured for nearly all enrolled participants, where during initial assessment information was collected on basic lifestyle and health information, anthropometric measurements, blood and urine samples, body composition, as well as a wealth of additional features. The large amounts of available measurements, taken independent of clinical indication, make the UKB particularly suited for a “hypothesis-free” data-driven approach to potentially identify predictors especially important for people with diabetes.

The current study aimed to uncover features for the 10-years risk of CVD in people with T2DM (w T2DM) and with T2DM and CVD (w T2DM&CVD), which can be used to improve attempts at early identification of high-risk individuals and help with the management of CVD. Our objective was to assess a larger number of features than typically considered in CVD risk prediction models, providing a comprehensive overview of key features that can, in turn, be used to inform the development of de novo models or to enrich existing CVD prediction models, tailoring them for people with type 2 diabetes with or without established CVD. To achieve this, we crafted an integrated data engineering and feature selection pipeline to identify the subset of 600 + UKB measured feature which are predictive of the onset of CVD during a 10-year follow-up period.

Analyses were conducted in three distinct groups of participants based on their clinical risk of CVD: without a history of diabetes or CVD at enrolment (T2DM/CVD), with T2DM at enrolment, and with T2DM&CVD at enrolment.

In this study, we show that classical risk factors are less important for predicting CVD in people with T2DM. We uncover and replicate numerous predictors which are relatively more important in people with diabetes, including mental health, familial CVD history, markers of ill health, kidney disease, and diabetes control, with some (e.g., glycated haemoglobin, cystatin C) predicting CVD regardless of diabetes status. Diabetes-specific features include dietary patterns (e.g. fruit consumption, dietary variability, or poultry or oily fish consumption) and biochemistry measures (e.g. alanine aminotransferase, rheumatoid factor, haematocrit percentage, or monocyte counts). Consideration of these non-traditional CVD risk factors in guideline recommended prediction models may improve their performance in people with diabetes.

## Methods

### Data source

Data was sourced from the UKB, a cohort of ~500,000 men and women aged 40–69 years between 2006 and 2010 enrolled from primary care registers across the UK^10^. The UK Biobank has ethical approval from the North West Multi-centre Research Ethics Committee to handle human participant data, no additional ethical approval was required because the study involved the secondary use of data. Written informed consent was obtained from all participants and all data is deidentified for analysis. Eligible researchers may access UK Biobank data on www.ukbiobank.ac.uk upon registration. This study was approved under UK Biobank Resource application numbers 12113, 24711 and 44972.

Th UKB data were stratified into three groups: people without a diagnosis of CVD or T2DM at enrolment (wo T2DM/CVD), the second group included participants with T2DM diagnosis but no history of CVD at enrolment (w T2DM,), and the final group included individuals with diabetes and a history of CVD at enrolment (w T2DM&CVD). Follow-up considered the time from enrolment until the first CVD event, death or end of the study (10-years after enrolment), whichever came first; see Supplementary Table 1. The candidate predictors were measured at the time of enrolment.

To identify features associating with the 10-years risk of CVD, we extracted variables (data fields) from 31 distinct UKB categories. The selected fields considered a range of information including anthropometry, blood chemistry, questionnaire data, and sociodemographic characteristics, jointly consisting of 603 unique variables; see Supplementary Table 2. Please see Supplementary Methods, Supplementary Figs. 1–3, Supplementary Tables 3–6, and Supplementary Data 1 for an overview of the sourced data and applied data engineering strategy.

### Statistics and reproducibility

After randomly splitting the UK Biobank data into 80% for training, and 20% for testing, the training data were used to prune data on multicollinearity (Spearman’s correlation ≥ ±0.70) and absence of an outcome associations (univariable *p*-value ≥ 0.80).

To identify CVD-related features we leveraged a generalized linear model with a binomial distribution and an elastic net penalty^11^ (combining L1 and L2 regularisation), seamlessly removing less important features^11^. Ten-fold cross-validation, stratified by case (people who developed CVD) and control status (people who did not develop CVD), was used to optimize model hyper-parameters. Given the substantial differences in subgroup sizes, models were separately trained for each participant subgroup and outcome, enabling the identification of features distinct to individuals with or without diabetes.

The feature importance of each selected variable was evaluated by applying a permutation feature importance algorithm (10 permutations) to the test data. This method quantified the change in the c-statistic and allowed us to identify and remove features with a zero or negative feature importance, which indicates a failure to replicate the original association. Features were subsequently ranked by their c-statistic change, stratified by outcome type (CVD + AF + HF, CVD, CHD, HF, AF, Isch. Stroke) and a participant group (“wo T2DM/CVD”, “w T2DM”, “w T2DM&CVD”). By estimating the feature importance in the test data, we were able to identify replicated findings (features with positive importance), which were unaffected by any potential overfitting. We dropped features with a zero or negative feature importance in the test data, indicating a failure to replicate. As age and sex are well-known and dominant CVD risk factors, the main text focussed on the remaining features, noting these remaining features are conditionally independent of age and sex; see full results in Supplementary Data 2.

To assess the extent of variation in feature importance rankings, we calculated the difference between diabetes groups and the non-diabetes group using the Wilcoxon statistical test. Next, we examined the importance of traditional CVD risk factors in relation to our identified non-traditional risk factors. For this purpose we determined the rank of features used in any the following three clinically used prediction models: ASCVD^12^, QRISK3^4^ and the Framingham 1998^13^ score. Additionally, to examine the contribution of lipid lowering medication on the overall study results, particularly on the low-density lipoprotein cholesterol (LDL-C) rank, we retrained the elastic net models using the training dataset and stratified by baseline use of lipid lowering medication.

Furthermore, to investigate non-linear associations, we conducted a random forest analysis for each participant subgroup-outcome combination. The ranked random forest-based feature importance, which quantifies the change in the c-statistic, was compared to the ranked feature importance from the elastic net model using Spearman’s correlation coefficients.

To ensure the results are reproducible, the manuscript, including Supplementary detail provide a comprehensive description of the data engineering and statistical analysis employed, with reproducibility further exchanged by sharing the codebase used for the described analyses^14^.

Analyses were carried out in Python v3.6 using *scikit-learn*^15^, *statsmodels*^16^, *pandas*^17^, and *numpy*^18^, plots were generated using *matplotlib*^19^, and *seaborn*^20^, imputation was performed in R v4.1 using *mice*^21^.

### Reporting summary

Further information on research design is available in the Nature Portfolio Reporting Summary linked to this article.

## Results

Our findings reveal that classical CVD risk factors, including LDL-C, are less important in people with diabetes. Depending on the history of CVD the most important predictors for people with diabetes but without a history of CVD included: cystatin C, self-reported health satisfaction, biochemical measures of ill health (e.g. plasma albumin). For people with diabetes and a history of CVD top features included self-reported ill health, and blood cell counts measurements (e.g. red cell distribution width). CVD risk factors specific to people with diabetes included information on dietary patterns, mental health, and biochemistry measures such as oestradiol, rheumatoid factor, and monocyte count.

### Participant characteristics

Data were available on 459,142 participants without T2DM and CVD at baseline “wo T2DM/CVD”, 14,610 individuals with T2DM but without a history of CVD at the time of diagnosis “w T2DM”, and 4,432 participants who had a history of CVD at the time of T2DM diagnosis “w T2DM&CVD”. Participants with CVD were on average older, male, had a higher BMI, and higher HbA_1c_ concentrations; see Table 1. During a median follow-up time of 10 years, 40,350 (8.8%) of the “wo T2DM/CVD” participants experienced a CVD + HF + AF event, with 2,671 (18.3%) CVD + HF + AF events in the “w T2DM” group, and 3,453 (77.9%) of the “w T2DM&CVD” group; Supplementary Table 7.

**Table 1:** Clinical characteristics of UK biobank participants stratified by history of CVD and T2DM at enrolment

	wo T2DM/CVD (individuals without a history of T2DM and CVD)		w T2DM (individuals with T2DM but without a history of CVD)		w T2DM&CVD (individuals with a history of T2DM and CVD)	
Clinical characteristic	Mean (SD) or *N* (%)	Missing data (%)	Mean (SD) or *N* (%)	Missing data (%)	Mean (SD) or *N* (%)	Missing data (%)
Total no. of individuals	459,142		14,610		4432	
Women (%)	258,250 (56.2)	0	6072 (41.6)	0	1191 (26.9)	0.0
Age (years)	56.2 (8.1)	0	59.0 (7.4)	0	62.2 (5.8)	0.0
Smoking Status:		3.3		3.6		4.2
Never	248,481 (54.1)		6956 (47.6)		1438 (32.4)	
Previous	149,525 (32.6)		5593 (38.3)		2250 (50.8)	
Current	46,073 (10.0)		1529 (10.5)		557 (12.6)	
BMI (kg/m2)	27.2 (4.6)	2.9	31.4 (5.8)	2.2	31.8 (5.6)	2.9
C-reactive protein (mg/L)	2.5 (4.2)	7.5	3.6 (5.2)	7.7	4.0 (6.1)	8.6
Creatinine (umol/L)	71.7 (16.0)	7.2	73.7 (24.3)	6.9	85.1 (41.9)	7.4
HDL cholesterol (mmol/L)	1.5 (0.5)	13.5	1.2 (0.4)	9.7	1.1 (0.3)	10.1
Total cholesterol (mmol/L)	5.8 (1.1)	7.2	4.6 (1.1)	6.8	4.3 (1.0)	7.3
LDL cholesterol (mmol/L)	3.6 (0.8)	6.9	2.8 (0.8)	5.9	2.5 (0.7)	6.0
HbA1C (mmol/mol)	35.3 (4.8)	7.7	54.7 (14.0)	3.5	54.9 (14.2)	3.8
SBP (mm Hg)	139.6 (19.7)	2.8	144.2 (18.2)	2.7	142.1 (20.2)	2.9
DBP (mm Hg)	82.3 (10.7)	8.0	82.5 (10.2)	7.0	78.1 (11.0)	7.6
Lipid lowering medication usage	20,933 (4.6)	0.0	5116 (35.0)	0.0	1875 (42.3)	0.0
Antihypertensive medication usage	42,893 (9.3)	0.0	4376 (30.1)	0.0	1853 (41.8)	0.0

Participant subgroups: people without diabetes or a history of CVD at enrolment (“wo T2DM/CVD”), people with diabetes but without a history of CVD at enrolment (“w T2DM”), and Clinical information was obtained from the UKB assessment centre data, and in case of missing information, data was extracted from the longitudinal GP records, selecting the measurements closest to baseline from within a time window of 1 year before and 3 months after baseline. SD refers to standard deviation.

*BMI* body mass index, *HDL cholesterol* high-density lipoprotein cholesterol, *LDL cholesterol* low-density lipoprotein cholesterol, *HbA1C* glycated haemoglobin, *SBP* systolic blood pressure, *DBP* diastolic blood pressure.

### Prioritized features for CVD prediction

Out of the 603 initially available UKB data fields, 382 were retained after the data engineering steps, depending on the type of CVD and participant subgroup between 229 and 258 features remainder after filtering on univariable association and multicollinearity; see Supplementary Table 8, Supplementary Fig. 4. An elastic net algorithm was applied to identify a subset of variables associated with CVD outcomes, which were subsequently replicated in the independent test set, resulting in a range of replicated features between 32 (for CHD in “w T2DM”) and 200 (for Isch. Stroke in “w T2DM&CVD”); Supplementary Fig. 4. Generally, our pipeline identified the most features for the wo T2DM/CVD subgroup (on average 156 features were replicated across the six considered CVD outcomes), followed by w T2DM&CVD (an average of 126 features), and w T2DM (an average of 63 features); Supplementary Fig. 4.

**Fig. 1:**
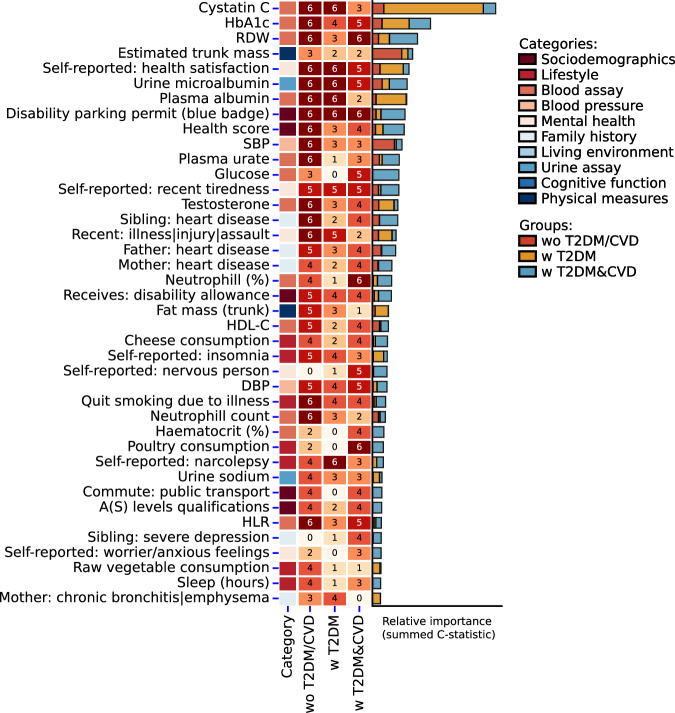
Top 40 most predictive features for the 10-years risk of cardiovascular disease. The y-axis presents the top 40 features (excluding age and sex) based on the summed feature importance aggregated across the six types of CVD considered (CVD **+** AF **+** HF, CVD, CHD, Ischemic Stroke, HF, AF) stratified by participants subgroup: people without diabetes or a history of CVD at enrolment (“wo T2DM/CVD”), people with diabetes but without a history of CVD at enrolment (“w T2DM”), and people with a history of diabetes and CVD at enrolment (“w T2DM&CVD”). Features were colour-coded by the UK Biobank defined category. The middle heatmap represents the number of CVD outcomes for which a specific feature was identified for (at most 6), while the stacked bar chart encodes the summed feature importance, stratified by participant subgroup. Feature importance was calculated using a permuted feature importance algorithm recording the change in c-statistic. The algorithm was applied to the hold-out test data and hence represent an unbiased estimates of the feature importance as well as reflecting features which were independently replicated. The complete list of identified features is provided in Supplementary Data 2. T2DM type 2 diabetes, CVD cardiovascular disease, RDW red blood cell distribution width, HbA1c glycated haemoglobin, SBP systolic blood pressure, HDL-C high-density lipoprotein cholesterol, DBP diastolic blood pressure, HLR high light scatter reticulocyte count.

Ranking features on their summed c-statistic, aggregated across CVD outcomes, (Fig. 1, Supplementary Data 2) highlighted the importance of plasma biomarkers such as cystatin C, red blood cell distribution width (RDW), HbA_1c_, plasma albumin, plasma urate, glucose, testosterone, and urine microalbumin, as well as clinical characteristics such as diastolic and systolic blood pressure (DBP/SBP), and estimated trunk mass. Additionally, many of the top ranking features included indicators of a poor health status (e.g., “self-reported: health satisfaction”, “quit smoking due to illness”, “self-reported: recent tiredness”, “disability parking permit (blue badge)”), family (father, mother, sibling) history of heart disease. While systolic/diastolic blood pressure (SBP/DBP) and high-density lipoprotein were ranked 12th, 28th, and 24th respectively, LDL-C conveyed relatively limited discriminative ability, ranking 112th. The results of the retrained elastic net models, stratified by the use of lipid-lowering medication, confirmed the limited importance of LDL-C for CVD prediction in diabetes subgroups, with LDL-C only being selected for the “w T2DM&CVD” group without initial lipid-lowering medication, where it was ranked 70th. LDL-C demonstrated better discriminative ability in the general population, ranking 32nd and 13th for individuals with and without lipid lowering medication, respectively.

The comparison of the coefficient and c-statistic feature rankings for CVD showed relatively strong agreement, with correlation coefficient of 0.90 (*p*-value ≈ 7.23 × 10^−33^) for “wo T2DM/CVD”, 0.76 (*p*-value ≈ 5.80 × 10^−5^) for “w T2DM”, and 0.60 (*p*-value ≈ 7.19 × 10^−6^) for “w T2DM&CVD”; see Supplementary Table 9 and Supplementary Data 3.

The comparison between features identified by the elastic net models and those identified by non-linear model, such as random forest models (Supplementary Tables 10–13) revealed a relatively high overlap between the top 40 features identified across all subgroups and outcomes. These features mainly included blood and urine assays, blood pressure, and physical measures; see Supplementary Fig. 5. The Spearman’s correlation between feature importance ranks from elastic net models and random forest models ranged between 0.59 and 0.69 for the “wo T2DM/CVD”, 0.41 and 0.69 for the “w T2DM” group, and 0.29 and 0.82 for the “w T2DM&CVD”; Supplementary Table 14. Please see Supplementary Data 4 for the random forest based feature importance for each participant subgroup and outcome.

### The top 5 most relevant features per CVD type and participant subgroup

**Fig. 2:**
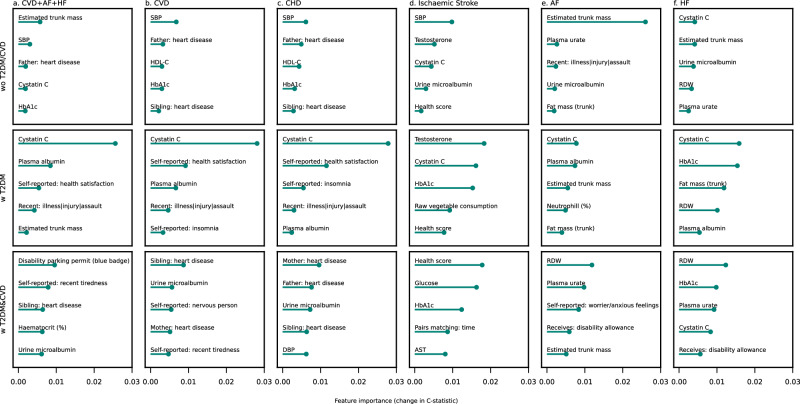
Top five most predictive features for the 10-years risk of cardiovascular disease, stratified by disease type. The y-axis presents the top five features (excluding age and sex), ranked by their feature importance, as shown on the x-axis. Results are stratified by type of CVD (**a**. CVD + AF + HF; **b**. CVD; **c**. CHD; **d**. Ischemic Stroke; **e**. AF; **f**. HF) and participant subgroup: people without diabetes or a history of CVD at enrolment (“wo T2DM/CVD”), people with diabetes but without a history of CVD at enrolment (“w T2DM”), and people with a history of diabetes and CVD at enrolment (“w T2DM&CVD”). Feature importance was calculated using a permuted feature importance algorithm recording the change in c-statistic. The algorithm was applied to the hold-out test data and hence represent an unbiased estimates of the feature importance as well as reflecting features which were independently replicated. The source data for this figure is provided in Supplementary Data 2. T2DM type 2 diabetes, CVD cardiovascular disease, CHD coronary heart disease, AF atrial fibrillation, HF heart failure, Isch. Stroke ischemic stroke, SBP systolic blood pressure, HDL-C high-density lipoprotein cholesterol, HbA1c glycated haemoglobin, RDW red blood cell distribution width, DBP diastolic blood pressure, disability parking permit (Receives: blue badge), AST aspartate aminotransferase.

The top 5 most important features per CVD type and participant subgroup is presented in Fig. 2, with the full list of features presented in Supplementary Data 2, and Supplementary Figs. 6–8. For people without T2DM or CVD at enrolment (wo T2DM/CVD), SBP and family history of heart disease were important features for CVD + AF + HF, CVD, and CHD, while HDL-C and HbA_1c_ were particularly important for CVD and CHD. Cystatin C and urine microalbumin populated the top 5 most important predictors for ischaemic stroke as well as HF, while estimated trunk mass was ranked highest for CVD + AF + HF and HF; Fig. 2.

For people with T2DM at the time of enrolment (w T2DM), Cystatin C was particularly important to predict all 6 CVD outcomes, with a feature importance of 0.028 for CHD in the “w T2DM” group, compared to only 0.001 in people without diabetes. Self-reported health satisfaction and self-reported insomnia were important for CVD + AF + HF, CVD, and CHD, where self-reported insomnia was also included as a top 5 predictor for CVD and CHD. HbA_1c_ was a strong predictor for Ischaemic stroke and HF, while fat mass and plasma albumin were important for AF and HF; Fig. 2 and Supplementary Data 2.

For people with T2DM and CVD at the time of enrolment (w T2DM&CVD), indicators of, sometimes recent, adversity in (perceived) health or stress were important risk factors for CVD. For example, owning a disability parking permit (CVD + HF + AF, HF), self-reported nervousness (CVD), self-reported worrier/anxious feelings (AF), or receiving a disability allowance (HF). Furthermore, familial history of heart disease was often included in the top 5 features for CHD + AF + HF, CVD, and CHD. More traditional biomarkers/clinical measurements were also retained in the top 5: urine albumin (CVD + AF + HF, CVD, CHD), DBP (CHD), haematocrit (CVD + AF + HF), RDW (AF, HF), plasma urate (AF, HF), glucose (ischaemic stroke) and HbA_1c_ (ischaemic stroke, HF); see Fig. 2 and Supplementary Data 2.

Finally, as detailed in Supplementary Data 2, we note that while age and sex were often the most important predictors irrespective of a participants diabetes status, in people with diabetes the c-statistic was severely attenuated compared to people without diabetes. For example, for CVD + AF + HF the c-statistic for age was 0.083 in the wo T2DM/CVD group, compared to 0.041 in the w T2DM group.

### CVD features selected in all three participants groups

We next identified features that were selected for all three participants groups, stratifying by CVD outcome type; Supplementary Data 5 and Supplementary Results. The number of common features ranged from 14 for CHD to 20 for AF. Briefly, we observed that HbA1c was an important predictor irrespective of the diabetes status, particularly for CVD, HF, AF, and Is. stroke. Interestingly, HbA1c was the 6^th^ most important predictor for CVD and CHD in people without diabetes, for people with diabetes HbA1c was selected for CVD, HF, and Isch. Stroke prediction. Cystatin C and RDW were predictive of HF, AF and Is. stroke (cystatin C only) irrespective of the participant subgroup. Aside from these biochemistry measures, we observed that information on familial disease history, self-reported health (satisfaction), mental-health and socio-economic factors were often predictive of CVD irrespective of the diabetes status.

### CVD features unique for people with T2DM

**Fig. 3:**
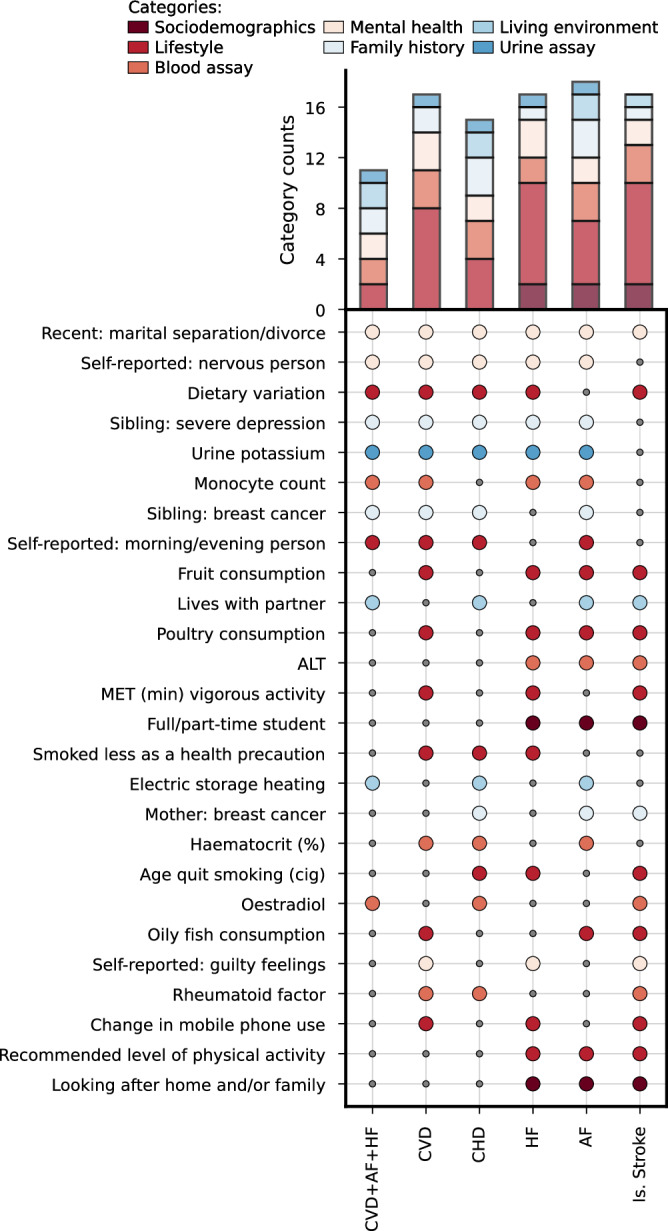
Diabetes specific features for the 10-years risk of cardiovascular disease. The figure focuses on features which were predictive for at least three of the six considered CVD outcomes, the complete list of features is presented in Supplementary Data 4−6. The large coloured dots in the middle panel indicate which diabetes-specific feature was selected for each outcome, with the dot colours representing the categories assigned to the features by the UK Biobank. The small grey dots in the middle panel indicates the feature was not selected for the specific outcome. The stacked bar chart represents the number of selected features grouped by UK Biobank assigned categories. T2DM type 2 diabetes, CVD cardiovascular disease, CHD coronary heart disease, AF atrial fibrillation, HF heart failure, Isch. Stroke ischemic stroke, MET metabolic equivalent task, ALT alanine aminotransferase. The top panel reflect the number of times feature belonging to a specific UK biobank category was selected.

Given the poor performance of CVD prediction models in people with T2DM, we next identified the union of features which were uniquely selected for “w T2DM” or “w T2DM&CVD” participant subgroups*;* Fig. 3, Supplementary Table 15, Supplementary Data 6−8. On average 5 lifestyle factor were unique to CVD prediction in people with diabetes, were particularly diet related information such as fruit consumption, dietary variability, or poultry or oily fish consumption l was paramount. Information on mental health (on average 2 features) and blood assay (on average 3 features) were also important for CVD prediction in people with diabetes. For example, self-reported nervousness, or severe depression, guilty feelings, and recent divorce or separation were all relevant and unique to CVD prediction in people with diabetes. Similarly, alanine aminotransferase, rheumatoid factor, haematocrit percentage, monocyte counts, and oestradiol were important and unique predictors for CVD in people with diabetes (Fig. 3). The difference in ranked feature importance between individuals with and without diabetes, determined for each combination of participant subgroups and type of CVD, confirmed significant differences for most combinations, except for AF and ischaemic stroke in “w T2DM”, and HF in “w T2DM&CVD”; see Supplementary Fig. 9.

### Ranking features used by three clinical CVD prediction models

We determined the importance and rank of 18 features included in at least one of the clinically used prediction models: ASCVD^12^, QRISK3^4^, and the Framingham^13^, Supplementary Tables 16−18. In people without diabetes (“wo T2DM/CVD”) 9 features were selected to predict CVD, with 7 in the top 10% (in order of importance: age, sex, SBP, paternal history of CVD, HDL-C, maternal history of CVD, and sibling history of CVD); Supplementary Table 16. For the “w T2DM” participants, 6 features were selected for CVD, and only age and sex were retained in the top 10 most important features; Supplementary Table 17. For “w T2DM&CVD” participants 8 features were selected for CVD, where age, sex, and maternal and sibling history of CVD were the top 10 most important features; Supplementary Table 17.The following features were not selected for either diabetes subgroup (“w T2DM”, or “w T2DM&CVD”): LDL cholesterol, total cholesterol, smoking status, BMI, severe mental illness, and ethnicity; Supplementary Tables 17–18. Finally, LDL-C was ranked 26.03% for people without diabetes, and not selected for people with diabetes irrespective of CVD history at enrolment, or baseline use of lipid-lowering medication, except for the “w T2DM&CVD” without initial intake of lipid-lowering medication, where LDL-C was ranked 70 in stratified analysis using retrained models.

### Benefit of considering the identified non-traditional CVD risk factors

We additionally estimated how many cases were appropriately assigned a higher risk by additionally considering information from the here identified non-classical risk factors. For this we compared risk group assignment (using the canonical risk groups <10%, between 10% and 20%, and ≥20%) based on the 18 classical risk factors used in the ASCVD^12^, QRISK3^4^, or the Framingham^13^ against risk group assignment combining classical and non-classical risk factors. Per 1,000 people without diabetes (the “wo T2DM/CVD” group) 253 participants who went on to develop CVD appropriately received a higher risk, for HF this was 36. Per 1,000 people with diabetes (the “w T2DM” group) these numbers were 133 for CVD and 165 for HF.

## Discussion

In this study, we leveraged data from the richly phenotyped UKB to identify potential predictors for CVD, specifically focusing on people with diabetes. Combining a bespoke data-engineering strategy with supervised feature selection using an elastic net algorithm, we found a prioritized list of 32 to 200 features, depending on the type of CVD considered. The classical risk factors (e.g. parental or maternal history of heart disease, and blood pressure) were relatively highly ranked for people without diabetes (“wo T2DM/CVD”), however, truncal mass was selected instead of BMI, and HDL-C rather than total cholesterol or LDL-C.

These traditional predictors were much less important in people with diabetes. Instead the following features were import to predict CVD in people with diabetes but without a history of CVD (“w T2DM”): HbA1c, cystatin C, self-reported health satisfaction, biochemical measures of ill health e.g. plasma albumin (representing liver and kidney damage). In people with diabetes and a history with CVD important features to predict CVD consisted of: self-reported ill health, and biochemical measures of ill health (RDW, haematocrit – representing anaemia) were selected. We additionally identified features common between people with and without diabetes. For example, HbA1c was important for people with and without diabetes to predict CVD and HF. Interestingly, HbA1c was important for predicting CVD and CHD in people without diabetes, ranking 6th. Additionally, features related to mental health (e.g., self-reported health satisfaction), disability status, urine microalbumin, and family disease history were common predictors for HF, CHD, and CVD. Focusing on features unique to people with diabetes highlighted the importance of information on diet variation (e.g. fruit or poultry consumption), physical activity, mental health (e.g., guilty feelings, nervousness), socioeconomic status (e.g., full/part-time student), family disease history (e.g., mother’s cancer), as well as blood assay measurements such as monocyte count, ALT, haematocrit, oestradiol, and rheumatoid factor.

The presented results suggest there is a need to consider additional, non-traditional, risk factors to identify people with diabetes at high-risk of CVD for early detection and treatment of CVD. Importantly, a substantial number of these risk factors may already be registered by clinicians (e.g. general practitioners) during their standard family and clinical history examination (e.g. on family disease, life events, employment). Other features may be readily obtained through questions focussing on diet and living environment, or by applying more bespoke anthropometrics focussing on truncal mass instead of BMI for example. Some features may require further consideration of costs and logistics, such as the need for additional lab measurements like cystatin c. The features identified in this study can be used to guide the development of new models or enhance existing CVD prediction models, specifically tailoring them for individuals with type 2 diabetes.

Due to the unique design of the UK Biobank, where measurements are obtained from all participants, irrespective of potential clinical diagnosis, we were able to highlight the relevance of kidney and diabetes markers for CVD prediction in people without such an indication. Importantly, these features were typically more discriminative than lipid measurements, suggesting that currently available risk prediction tools for CVD might be further optimized by adding early markers for kidney disease and diabetes. This held true for people with and people without diabetes, for example HbA1c was in the top 5% of most important CHD predictors for people without diabetes. The observations from this study, indicating the limited predictive ability of LDL-C, align with previous reports^22^. Notably, popular risk scores such as QRISK3^4^ and Framingham^9^ do not consider LDL-C as a required predictor), and most CVD models include HDL-C or TG instead; for instance, out of 22 CVD risk prediction models considered by Dziopa et al. 2021, only a single model did not incorporate HDL-C^3^. This of course does not imply that LDL-C does not have a causal effect on CHD or CVD, instead this highlights that good predictors of disease risk need not have a causal effect on disease manifestation. This adage is also exemplified by the selection algorithm including features, such as sex, maternal and paternal disease history, educational attainment or socioeconomic status.

While some of the here reported features have previously been associated with CVD and its individual components such as CHD, ischemic stroke, AF and HF, the current study is able to uniquely account for their pairwise correlation, thereby ensuring the identified features provide *independent* information. Additionally, by training a multivariable model we were able to estimate relative feature importance, and thereby rank features on their relevance for disease classification. Such a rank provides directly actionable information for clinicians and researchers wishing to enrich the currently available prediction algorithms. Furthermore, by identifying features for six types of CVD, we observe that many of the identified features are shared by distinct diseases such as CHD, HF, and AF. The commonality between features suggests that the same or similar information can be used to predict multiple types of CVD and hence better inform participant risk and optimize care, further supporting our previous study which generalized CVD risk prediction tools to predict the 10-years risk of HF and AF^3^. Importantly, we wish to clarify the communality is not the result of applying a single machine learning algorithm to model all disease across the three participant groups. Our analyses were specifically designed to maximise flexibility by employing independent models and feature selection processes for each participant group and outcome combination.

This study has some limitations which warrant discussion. Firstly, the UKB predominantly consists of white European participants, with an on average higher socioeconomic status, and hence generalizability to other ethnicities should be explored. Secondly, some of the identified features might be specific to the UK, for example, a Blue Badge is the UK’s version of a disabled parking permit. We do expect that many of these UK-specific features can be implemented in different countries given sufficiently careful mapping, similar to how laboratory measurement might need to be recalibrated between distinct labs. Thirdly, we emphasize that the identified features, feature importance, and feature rank were based on the testing data and therefore are unaffected by any potential model overfit and at the same time represents independently replicated findings. Nevertheless, with increasing sample size additional features will likely be identified, particularly for relatively infrequent outcomes such as Is. Stroke. Aside from the influence of sample size, due to the multivariate nature (where features are correlated among themselves), similar but slightly different features might be identified in subsequent studies (e.g., SBP may stand in for DBP due to the correlation between both measurements). While non-linear associations are to be expected in health and healthcare, often simplified models such as applied here are sufficient to detect the presence of an association. A follow-up analysis exploring potential non-linear relationships by applying a random forest algorithm, revealed a relatively high overlap among the top 40 most important features identified by random forest and elastic net models was relatively high (e.g. above 0.60). However, we observed reduced correlations for the “w T2DM&CVD” group, which may indicate an increased importance of non-linear associations, or could also reflect the smaller sample size, as well as the fact that, unlike elastic net algorithms, random forest does not actively remove features through penalisation.

In this scaled analysis of the UK Biobank, we showed that the classical risk factors were less importance in people with diabetes. We have identified numerous independent variables which predict CVD in people with diabetes, covering information on mental health, familial CVD and non-CVD disease histories, as well as markers for general ill-health, early kidney disease and diabetes (control). We note that some of the identified features, such as HbA1c and cystatin C, predict CVD irrespective of the diabetes status. Furthermore, we identified diabetes specific features predicting CVD typically covering dietary patterns, mental health and biochemistry measures. The identified features are typically overlooked by currently available CVD prediction models and provide actionable leads to improve CVD prediction models.

## Supplementary information

Supplementary Information

Description of Additional Supplementary Files

Supplementary Data 1

Supplementary Data 2

Supplementary Data 3

Supplementary Data 4

Supplementary Data 5

Supplementary Data 6

Supplementary Data 7

Supplementary Data 8

Supplementary Software

Supplementary Software Information

Reporting Summary

## Data Availability

The UK Biobank dataset analysed during the current study is available via the UK Biobank data access process (see http://www.ukbiobank.ac.uk/register-apply/). Detailed information about UK biobank categories and data fields is available at https://biobank.ctsu.ox.ac.uk/crystal/browse.cgi and https://biobank.ctsu.ox.ac.uk/ukb/help.cgi?cd=data_field. The source data for Figs. 1–2 are provided in Supplementary Data 2, and for Fig. 3 in Supplementary Data 8.

## Abbreviation

AF: Atrial fibrillation
BMI: Body Mass Index
CHD: Coronary heart disease
CVD: Cardiovascular disease
CVD + HF + AF: Cardiovascular disease including heart failure and/or atrial fibrillation
DBP: Diastolic blood pressure
HDL-C: High-density lipoprotein cholesterol
HF: Heart failure
LDL-C: Low-density lipoprotein cholesterol
MI: Myocardial infarction
SBP: Systolic blood pressure
T2DM: Type 2 diabetes
UKB: UK Biobank
w T2DM: Individuals with T2DM diagnosis but not history of CVD
w T2DM&CVD: Individuals with T2DM and a history of CVD
wo T2DM/CVD: Individuals without T2DM and a history of CVD

## Code availability

See 10.5281/zenodo.14779981^14^ for the codebase underpinning this work.
