# Supplementary material for: Identifying and ranking non-traditional risk factors for cardiovascular disease prediction in people with type 2 diabetes
Source: Commun Med (Lond). 2025 Mar 14;5:77. doi: 10.1038/s43856-025-00785-y (PMC11906859; doi:10.1038/s43856-025-00785-y)
Supplement: Supplementary file 1 — Supplementary Information [file 43856_2025_785_MOESM1_ESM.pdf]

Identifying and ranking non-traditional risk factors for cardiovascular disease prediction in people with T2DM

Katarzyna Dziopa, Nishi Chaturvedi, Folkert W Asselbergs, Amand F Schmidt

Contents

1 Supplementary Information **9**

1.1 Supplementary Methods 9

1.1.1 Outcome definitions 9

1.1.2 UK Biobank structure 9

1.1.3 Data engineering strategy 10

1.1.4 Statistical analysis 12

1.1.5 Identification of mutually independent features 13

1.1.6 Feature importance 13

1.2 Supplementary Results 14

1.2.1 CVD features selected in all three participants groups 14

1.2.2 Difference in discriminative performance between training and testing samples 15

1.3 Supplementary Tables 15

1.4 Supplementary Figures 28

List of Figures

1 Overview of the study design pipeline. 29
2 Flow diagram of the initial filtering and standardization step. 30
3 Flow diagram of data type-specific transformation step. 31
4 Overview of features available at the different stages of our feature selection algorithm. 32
5 Top 40 most predictive features for the 10-years risk of cardiovascular disease identified by random forest models. 33
6 The contribution of the top features to the prediction of six facets of CVD for ”wo T2DM/CVD” group. 34
7 The contribution of the top 60 features to the prediction of six facets of CVD for ”w T2DM” group. 35
8 The contribution of the top 60 features to the prediction of six facets of CVD for ”w T2DM&CVD” group. 36
9 Results of Wilcoxon tests on the difference in ranked feature importance identified by elastic net models between ”wo T2DM/CVD” and ”w T2DM”, and between ”wo T2DM/CVD” and ”w T2DM&CVD.” 37

List of Tables

1 Outcome definitions based on the HDR UK Phenotype Library. . . . 16
2 UK Biobank category identifiers included in a study. 17
3 List of the UK Biobank data fields that were combined. 18
4 Identifiers of country-specific UK Biobank data fields 18
5 UK Biobank (UKB) question answers replaced by missing values. . 18
6 Average percentage of missing data for each UK Biobank categories after data engineering 19
7 The number of variables used for training a multivariable elastic net algorithm and the number of CVD events during a 10-year follow-up period stratified by training and testing samples. 19
8 Number of data fields excluded based data-driven filtering steps. . . 20
9 Comparison of feature importance ranks based on the elastic net regression coefficients and the c-statistics using Spearman's correlation coefficient. 21
10 Discrimination multivariable elastic net models predicting 10-years risk of six types of CVD. 22
11 Parameters configuration for exhaustive search over parameter values for an estimator for random forest models. 22
12 Optimal configuration for the random forest models across three subgroups: ”wo T2DM/CVD”, ”w T2DM”, and ”w T2DM&CVD” and six CVD outcomes, identified through an exhaustive search over parameter values for an estimator. 23

13 Discrimination multivariable random forest models predicting 10-years risk of six types of CVD. 24
14 Comparison of feature importance ranks identified by elastic net models and random forest models based on regression coefficients and c-statistics using Spearman's correlation coefficient. 25
15 The number of features for the 10-years risk of cardiovascular disease unique to people with diabetes, stratified by disease type. 26
16 Ranks of the Qrisk3, ASCVD, and Framingham variables identified in ”wo T2DM/CVD” group for CVD outcome. 26
17 Ranks of the Qrisk3, ASCVD, and Framingham variables identified in ”w T2DM” group for CVD outcome. 27
18 Ranks of the Qrisk3, ASCVD, and Framingham variables identified in ”w T2DM&CVD” group for CVD outcome. 27

Supplementary Information

1.1 Supplementary Methods

1.1.1 Outcome definitions

CVD was defined as the occurrence of fatal or non-fatal myocardial infarction (MI), sudden cardiac death, ischemic heart disease, fatal or non-fatal stroke or PAD after the start of follow-up. We additionally considered a broader definition of CVD, also including heart failure (HF) and/or atrial fibrillation (AF): ”CVD+HF+AF”, as well as the individual CVD components: CHD, stroke, AF, and HF; see Supplementary Table 1.

1.1.2 UK Biobank structure

The data fields in the UK Biobank cover multiple time points, and within each time point, a variable maybe be recorded several times. For example, the severity of manic/irritable episodes (data field id = 5674 [1]) was recorded at four-time points: initial assessment visit, first repeat assessment visit, imaging visit, and first repeat imaging visit). The UK Biobank terminology defines each time point as an “instance”, and the measurements recorded at a single time point as “arrays”. The column format is defined as $field\_id - instance.array$ [2]. For example, column ”5674-0.0” refers to the initial assessment visit, while ”5674-1.0” refers to the first subsequent visit. Data field id = 5076 (”Number of letters correct in round (right)” [3]) presents an example where multiple measurements were recorded during a single visit e.g. ”5076-0.0”, ”5076-0.1”, ”5076-0.2”.

Variables types include continuous, integer, categorical (one possible answer), categorical (multiple possible answers), text, time, and compound[4]. Here compound describes a variable type where a set of variables are required to describe some compound property, for example, an applanation curve (describes the reflected light signal from the cornea, field id = 5266). A categorical (one possible answer) field could refer to a binary response (e.g., yes or no), but could also refer to multiple responses from which a participant could only select one. The categorical (multiple possible answers) fields allowed participants to provide multiple answers, for example, the response option to the question ”Which of the following do you never eat?” included ”eggs”, ”dairy products”, ”wheat products”, ”sugar”, ”I eat all of the above”, or ”Prefer not to answer”.

1.1.3 Data engineering strategy

We developed a de novo data engineering pipeline, and subsequently performed feature selection using an elastic net algorithm, where identified features were replicated in a 20% independent hold-out set using a permuted change in c-statistics to estimate feature importance. This feature selection pipeline was independently applied to six outcomes: coronary heart disease (CHD), ischemic stroke, heart failure (HF), atrial fibrillation (AF), CVD (combining CHD, stroke, and peripheral arterial disease (PAD)), and a broader definition of CVD+HF+AF, including HF and AF - outcomes which are more common in people with diabetes [5], [6].

The raw UKB data was curated using a purpose-built data-engineering pipeline returning quality-controlled tabular data allowing for subsequent analysis; see Supplementary Figure 1. Briefly, the data engineering procedure was split into two parts: 1) basic filtering and standardization: excluding meta-data, non-baseline features, and curating retained fields (e.g., combining information recorded in separate fields, see Supplementary Methods, Supplementary Figure 2), and 2) type-specific transformation: creating binary variables from categorical fields, removing invariant variables and categories with too few occurrences (Supplementary Methods, Supplementary Figure 3).

In the *initial filtering and standardization* (Supplementary Figure 2) fields

were excluded if they did not present data measured at enrolment. Additionally, pilot fields, representing a subset of the data available in the non-pilot data, and categorical fields with one possible answer, but with multiple data items measured at the same time were removed. Subsequently, data fields containing meta-data, such as time (representing time of measurement), text (representing information such as device type or id) were removed from the analysis. Given the often complex inter-relationship between compound fields, these were excluded from the analysis. Furthermore, country-specific variables not recorded for all considered countries (England, Scotland, Wales) were removed. The mean value was used for continuous (floating point numbers e.g. participant height) and integer (whole number e.g. participant age) fields with multiple measurements at the same assessment visit. Additionally, related data fields including data fields available for all considered countries were merged; see Supplementary Table 3 and Supplementary Table 4. For example, a single combine rule links all data fields that measure the participant's weight (data field ids: 3160 (manual entry) from body size measures, 23098 from body composition by impedance) or glucose (data field ids: 30740 from blood chemistry, 23470 from NMR spectroscopy). Next, 98 semantic rules (consisting of conditioning and affected data fields) were manually determined and reviewed (by KD and AFS) to mitigate the issue of missing responses wherever it was possible; see Supplementary Data 1. This is due to the nature of the UKB, where not all of the participants were asked the same set of questions, the follow-up question may depend on a participant's previous answer. The semantic rules were defined using a related field to determine a missing value. For example, data field id = 1249 ("Past tobacco smoking") was collected from participants except those who indicated they currently smoke on most or all days, as defined by their answers to data field = 1239 ("Current tobacco smoking"). Finally, procedural responses such as "Do not know" or "Prefer not to answer" were replaced by missing values (Supplementary Table 5).

The initial filtering step was followed by a *type-specific transformation* step (Supplementary Figure 3 adopted from [7]), where retained data was further processed. For example, continuous data were screened for a percentage of constant

values, where fields where more than 20% of participants had the same value, were additionally scrutinised to determine whether this information might be better represented as binary or categorical coding, or removed entirely. For questions where participants could provide multiple answers the categorical variable was converted to a set of binary indicator variables. For example, for the variable considering reasons of reduced smoking (data field = 6158 [8]), with values ”illness or ill health”, “doctor’s advice”, “health precaution”, or ”financial reasons”, I created binary variables e.g. ”illness or ill health”: $\{True, False\}$, or “doctor’s advice”: $\{True, False\}$, and so on.

While the UKB was designed to capture information about all participants, irrespective of possible clinical diagnoses, some of the data is affected by missing data. Furthermore, some of the data fields are only available (by design) for a subset of participants. This issue was partially addressed by the data engineering steps, where a part of the missing values was filled using semantic rules (see Supplementary Data 1). Additionally, features that were retained after the data engineering step were screened for the percentage of missingness, excluding categories (i.e., containing multiple variables) where over 40% of the values were missing; see Supplementary Tables 2, 6. The dataset was enhanced by basic demographic variables such as age, sex, and country of origin information.

1.1.4 Statistical analysis

In the current analysis, we exclusively focussed on measurements which were offered to all participants, with the limited number of missing data points imputed using the R package MICE [9].

After randomly splitting the data into 80% for training, and 20% for testing, the training data was used to prune features which showed a very weak univariable association with the considered outcomes. Specifically, for each of the 6 CVD outcomes we calculated the Spearman’s correlation and dropped variables with a p-value equal or larger than 0.80. Subsequently, we identified variables with an absolute pairwise Spearman’s correlation of 0.70 or larger – indicative of multicollinearity, dropping one of the variables that made up a multicollinear pair.

1.1.5 Identification of mutually independent features

To identify novel CVD-related features we leveraged a generalized linear model with a binomial distribution and an elastic net penalty (reflecting a combination of L1 and L2 penalty), removing features not associated with the considered outcome. Elastic net models will automatically perform a feature selection step, based on the required regularization necessary to optimize the c-statistic, where for the same number of candidate features but a smaller number of cases the expected regularization will increase (and hence the number of selected features will decrease) [10]. Ten-fold cross-validation, stratified by case (people who developed CVD) and control status (people who did not develop CVD), was used to optimize model hyper-parameters - e.g., to decide on the amount and type (the L1/L2 ratio) of penalization, using the available training data.

1.1.6 Feature importance

The feature importance of each selected variable was evaluated using a permutation feature importance algorithm (using 10 permutations), quantifying the change in the c-statistic. Features were subsequently ranked by their c-statistic change, stratified by outcome type (CVD+AF+HF, CVD, CHD, HF, AF, Isch. Stroke) and a participant group ("wo T2DM/CVD", "w T2DM", "w T2DM&CVD"). To provide an aggregate evaluation of the importance of each feature, we additionally recorded how often a feature was selected in each of the 6 available outcomes and calculated the summed c-statistic per participant group. The feature importance permutation algorithm was applied to the test data to provide independent replication, and as additional assurance against over-fitting. Here we dropped features with a zero or negative feature importance in the test data, indicating a failure to replicate. As age and sex are well-known and dominant CVD risk factors, the main text focussed on the remaining features, noting these remaining features are conditionally independent of age and sex. Performance of age and sex was included along with a complete list of the identified features in the relevant Supplementary Data 2.

Next, we identified the rank of features used in any the following three clinically

used prediction models: ASCVD [11], QRISK3 [12] and the Framingham 1998 [13] score. Here we focussed on predicting 10-years risk of CVD, differentiated between whether a variable included in these three models was selected by our feature selection pipeline, and if so what its rank across the three participant groups.

To illustrate the benefit of these novel risk factors for risk classification we calculated the net-reclassification index [14] for the onset of CVD or HF, comparing predictions based on the classical risk factors employed by the ASCVD [11], QRISK3 [12] and the Framingham 1998 [13] models, compared to consideration of both novel and classical risk factors. For these calculations we applied risk cut-offs of below 10% risk, between 10 and 20%, and above 20%, and focussed on the wo T2DM/CVD and w T2DM participant subgroups given that the participants of w T2DM/CVD predominantly received predicted risk above 20%. We calculated the number of cases which appropriately received a higher risk by calculating the difference between the proportion of cases receiving a higher risk and the proportion of cases receiving a lower risk, multiplying the difference by a 1000.

Despite this manuscript not necessarily focussing on deriving a novel prediction model, the models were evaluated in terms of their discriminative (c-statistic) ability using the independent test dataset. The differences in discriminate performance between training and testing were used as a metric of model over-fit, where an overfitted model reflects sample size-specific peculiarities, instead of generalizable findings. This complemented the feature importance estimates calculated on the independent test dataset, which by using the independent test set, were unaffected by any potential model overfit.

1.2 Supplementary Results

1.2.1 CVD features selected in all three participants groups

To show the commonalities between all three groups, we next identified features that were selected for all three participant subgroups, stratifying by CVD outcome type; Supplementary Data 5. The number of common features ranged from 14 for CHD to 20 for AF. For CHD the common features recorded information on familial

disease history (e.g., familial history of heart disease), self-reported general health (e.g. quit smoking due to illness, insomnia, narcolepsy), socio-economic factors (e.g. university degree), and biochemistry (e.g., HDL-C, urinary microalbumin). For AF common features included anthropometrics (e.g. estimated trunk mass, hip circumference), self-reported health (e.g. recent illness or injury), familial disease history (sibling with Parkinson's disease), biochemistry (e.g. plasma urea, urine microalbumin, total bilirubin, neutrophil percentage, cystatin C). The HF common features covered socioeconomic factors (e.g., "housing: renting/owning", unable to work due to illness), biochemistry (e.g. urate, Cystatin C, HbA1c, RDW), self-reported health (e.g. health satisfaction) and familial disease history (e.g. paternal history of dementia). For Ischemic stroke the common features included systolic blood pressure, biochemistry (e.g. HbA1c, Cystatin C, testosterone), diet (e.g. intake of raw vegetables, cheese, eggs, diary, and sugar), self-reported health (e.g. health satisfaction), and familial disease history (e.g. sibling history of prostate cancer).

1.2.2 Difference in discriminative performance between training and testing samples

The elastic net models were derived in a training and testing split, where performance in the test set provides an unbiased estimate of performance, unaffected by potential model over-fitting which would also impact feature selection. To identify potential problematic analyses, affected by over-fitting, we calculated the difference in c-statistic between the training and testing sets, where a large difference is indicative of model over-fit. The difference was smaller than 0.1 for all analyses, aside from a relatively large difference of 0.18 for the model predicting Ischaemic stroke in people with T2DM and CVD at enrolment, which correlated with a small number of cases (316) relative to the number of candidate predictors (238); see Supplementary Table 7, Supplementary Table 13.

1.3 Supplementary Tables

Supplementary Table 1: Outcome definitions based on the HDR UK Phenotype Library.

**Type of cardiovascular disease**	**Link to the HDR UK Phenotype Library**
Fatal and non-fatal MI	https://phenotypes.healthdatagateway.org/phenotypes/PH215/version/430/detail/<br>https://phenotypes.healthdatagateway.org/phenotypes/PH1027/version/2263/detail/
Fatal or non-fatal Stroke	https://phenotypes.healthdatagateway.org/phenotypes/PH55/version/110/detail/<br>https://phenotypes.healthdatagateway.org/phenotypes/PH56/version/112/detail/<br>https://phenotypes.healthdatagateway.org/phenotypes/PH85/version/170/detail/<br>https://phenotypes.healthdatagateway.org/phenotypes/PH86/version/172/detail/
Fatal peripheral vascular disease	https://phenotypes.healthdatagateway.org/phenotypes/PH236/version/472/detail/
Sudden Cardiac Death	https://phenotypes.healthdatagateway.org/phenotypes/PH995/version/2173/detail/
HF	https://phenotypes.healthdatagateway.org/phenotypes/PH1028/version/2265/detail/
AF	https://phenotypes.healthdatagateway.org/phenotypes/PH36/version/72/detail/
Ischemic stroke	https://phenotypes.healthdatagateway.org/phenotypes/PH56/version/112/detail/,<br>https://phenotypes.healthdatagateway.org/phenotypes/PH85/version/170/detail/
Haemorrhagic stroke	https://phenotypes.healthdatagateway.org/phenotypes/PH55/version/110/detail/,<br>https://phenotypes.healthdatagateway.org/phenotypes/PH86/version/172/detail/

Supplementary Table 2: UK Biobank category identifiers included in a study.

Category Id	Category description
100009	Body composition by impedance - Anthropometry - Physical measures - Assessment Centre
100010	Body size measures - Anthropometry - Physical measures - Assessment Centre
100014	Autorefraction - Eye measures - Physical measures - Assessment Centre
100015	Intraocular pressure - Eye measures - Physical measures - Assessment Centre
100017	Visual acuity - Eye measures - Physical measures - Assessment Centre
100019	Hand grip strength - Physical measures - Assessment Centre
100027	Fluid intelligence / reasoning - Cognitive function - Assessment Centre
100028	Lights pattern memory - Cognitive function - Assessment Centre
100030	Pairs matching - Cognitive function - Assessment Centre
100031	Prospective memory - Cognitive function - Assessment Centre
100032	Reaction time - Cognitive function - Assessment Centre
100034	Family history - Touchscreen - Assessment Centre
100052	Diet - Lifestyle and environment - Touchscreen - Assessment Centre
100053	Electronic device use - Lifestyle and environment - Touchscreen - Assessment Centre
100057	Sleep - Lifestyle and environment - Touchscreen - Assessment Centre
100058	Smoking - Lifestyle and environment - Touchscreen - Assessment Centre
100060	Mental health - Psychosocial factors - Touchscreen - Assessment Centre
100063	Education - Sociodemographics - Touchscreen - Assessment Centre
100064	Employment - Sociodemographics - Touchscreen - Assessment Centre
100065	Ethnicity - Sociodemographics - Touchscreen - Assessment Centre
100066	Household - Sociodemographics - Touchscreen - Assessment Centre
100067	Other sociodemographic factors - Sociodemographics - Touchscreen - Assessment Centre
100077	Word production - Cognitive function - Assessment Centre
100081	Blood count - Blood assays - Biological samples
100083	Urine assays - Biological samples
100099	Eye surgery/complications - Eye measures - Physical measures - Assessment Centre
114	Residential air pollution - Local environment - Additional exposures
17518	Blood biochemistry - Blood assays - Biological samples
54	MET Scores - Physical activity - Lifestyle and environment - Touchscreen - Assessment Centre
76	Indices of Multiple Deprivation - Baseline characteristics - Population characteristics
100011	Assessment centre - Physical measures - Blood pressure

Supplementary Table 3: List of the UK Biobank data fields that were combined.

Data field identifiers	Unified data field name
26414,26431,26421	Education score
26412,26429,26419	Employment score
26413,26430,26420	Health score
26415,26432,26423	Housing score
26411,26428,26418	Income score
26410,26427,26426	Index of Multiple Deprivation
3160,23098,21002	Weight
21001,23104	Body mass index (BMI)
4079,94	Diastolic blood pressure
95,102	Pulse rate
4080,93	Systolic blood pressure

Supplementary Table 4: Identifiers of country-specific UK Biobank data fields

Data field ids	Data field description
26414 (England), 26431 (Scotland), 26421 (Wales)	Education score
26412 (England), 26429 (Scotland), 26419 (Wales)	Employment score
26413 (England), 26430 (Scotland), 26420 (Wales)	Health score
26415 (England), 26432 (Scotland), 26423 (Wales)	Housing score
26411 (England), 26428 (Scotland), 26418 (Wales)	Income score
26410 (England), 26427 (Scotland), 26426 (Wales)	Index of Multiple Deprivation

Supplementary Table 5: UK Biobank (UKB) question answers replaced by missing values.

UKB response identifier	UBK description
-1	Question not asked due to previous answers
-3	Prefer not to answer
-7	None of the above
-11	Do not know (group 1)
-21	Do not know (group 2)
-1	Do not know
-1	Participant skipped/abandonded
-17	None of the above (group 1)
-23	Prefer not to answer (group 2)
-27	None of the above (group 2)
-5	Not sure
-13	Prefer not to answer (group 1)
-10	Less than once a week

Supplementary Table 6: Average percentage of missing data for each UK Biobank categories after data engineering .

Category Id	Category description	Average % missing
100009	Body composition by impedance - Anthropometry - Physical measures - Assessment Centre	2.05
100010	Body size measures - Anthropometry - Physical measures - Assessment Centre	14.72
100011	Assessment centre - Physical measures - Blood pressure	34.42
100014	Autorefraction - Eye measures - Physical measures - Assessment Centre	80.01
100015	Intraocular pressure - Eye measures - Physical measures - Assessment Centre	77.70
100017	Visual acuity - Eye measures - Physical measures - Assessment Centre	77.31
100019	Hand grip strength - Physical measures - Assessment Centre	0.67
100027	Fluid intelligence / reasoning - Cognitive function - Assessment Centre	78.99
100030	Pairs matching - Cognitive function - Assessment Centre	0.93
100031	Prospective memory - Cognitive function - Assessment Centre	65.88
100032	Reaction time - Cognitive function - Assessment Centre	1.49
100034	Family history - Touchscreen - Assessment Centre	7.04
100052	Diet - Lifestyle and environment - Touchscreen - Assessment Centre	4.49
100053	Electronic device use - Lifestyle and environment - Touchscreen - Assessment Centre	11.50
100057	Sleep - Lifestyle and environment - Touchscreen - Assessment Centre	3.10
100058	Smoking - Lifestyle and environment - Touchscreen - Assessment Centre	10.06
100060	Mental health - Psychosocial factors - Touchscreen - Assessment Centre	33.86
100063	Education - Sociodemographics - Touchscreen - Assessment Centre	5.62
100064	Employment - Sociodemographics - Touchscreen - Assessment Centre	7.12
100065	Ethnicity - Sociodemographics - Touchscreen - Assessment Centre	0.55
100066	Household - Sociodemographics - Touchscreen - Assessment Centre	24.60
100067	Other sociodemographic factors - Sociodemographics - Touchscreen - Assessment Centre	16.75
100081	Blood count - Blood assays - Biological samples	5.32
100083	Urine assays - Biological samples	4.09
100099	Eye surgery/complications - Eye measures - Physical measures - Assessment Centre	90.12
114	Residential air pollution - Local environment - Additional exposures	4.72
17518	Blood biochemistry - Blood assays - Biological samples	15.79
54	MET Scores - Physical activity - Lifestyle and environment - Touchscreen - Assessment Centre	19.70
76	Indices of Multiple Deprivation - Baseline characteristics - Population characteristics	13.90

Supplementary Table 7: The number of variables used for training a multivariable elastic net algorithm and the number of CVD events during a 10-year follow-up period stratified by training and testing samples.

Group	Outcome	Total sample size	Train sample size	Test sample size	No. events train (%)	No. events test (%)	No. variables
wo T2DM/CVD	CVD+AF+HF	459142	367313	91829	32306 (8.8)	8044 (8.8)	252
w T2DM	CVD+AF+HF	14610	11688	2922	2130 (18.2)	541 (18.5)	246
w T2DM&CVD	CVD+AF+HF	4432	3545	887	2745 (77.4)	708 (79.8)	236
wo T2DM/CVD	CVD	459142	367313	91829	22021 (6.0)	5447 (5.9)	255
w T2DM	CVD	14610	11688	2922	1619 (13.9)	415 (14.2)	239
w T2DM&CVD	CVD	4432	3545	887	2617 (73.8)	669 (75.4)	234
wo T2DM/CVD	CHD	459142	367313	91829	17394 (4.7)	4318 (4.7)	255
w T2DM	CHD	14610	11688	2922	1271 (10.9)	312 (10.7)	233
w T2DM&CVD	CHD	4432	3545	887	2464 (69.5)	639 (72.0)	228
wo T2DM/CVD	HF	459142	367313	91829	3985 (1.1)	969 (1.1)	251
w T2DM	HF	14610	11688	2922	405 (3.5)	91 (3.1)	224
w T2DM&CVD	HF	4432	3545	887	728 (20.5)	214 (24.1)	237
wo T2DM/CVD	AF	459142	367313	91829	15058 (4.1)	3745 (4.1)	257
w T2DM	AF	14610	11688	2922	915 (7.8)	221 (7.6)	235
w T2DM&CVD	AF	4432	3545	887	879 (24.8)	234 (26.4)	237
wo T2DM/CVD	Is. Stroke	459142	367313	91829	3588 (1.0)	841 (0.9)	251
w T2DM	Is. Stroke	14610	11688	2922	266 (2.3)	74 (2.5)	228
w T2DM&CVD	Is. Stroke	4432	3545	887	316 (8.9)	72 (8.1)	238

The UK Biobank participants were grouped based on T2DM and CVD histories at the time of enrolment: participants without a history of CVD and T2DM (wo T2DM/CVD), participants with type 2 diabetes (w T2DM), participants with a history of CVD before a T2DM diagnosis (w T2DM&CVD).

Supplementary Table 8: Number of data fields excluded based data-driven filtering steps.

Group	Outcome	Insufficient outcome correlation	Multicolinearity
wo T2DM/CVD	CVD+AF+HF	9	121
w T2DM	CVD+AF+HF	19	117
w T2DM&CVD	CVD+AF+HF	33	112
wo T2DM/CVD	CVD	5	122
w T2DM	CVD	28	115
w T2DM&CVD	CVD	42	105
wo T2DM/CVD	CHD	7	120
w T2DM	CHD	34	115
w T2DM&CVD	CHD	35	118
wo T2DM/CVD	HF	13	118
w T2DM	HF	46	111
w T2DM&CVD	HF	36	108
wo T2DM/CVD	AF	8	117
w T2DM	AF	36	111
w T2DM&CVD	AF	37	107
wo T2DM/CVD	Isch. Stroke	17	114
w T2DM	Isch. Stroke	43	111
w T2DM&CVD	Isch. Stroke	36	107

Supplementary Table 9: Comparison of feature importance ranks based on the elastic net regression coefficients and the c-statistics using Spearman's correlation coefficient.

Group Name	Outcome	Spearman's rank correlation	Spearman's p-value
wo T2DM/CVD	CVD+AF+HF	0.943315	7.516698e-46
wo T2DM/CVD	CVD	0.898553	7.228298e-33
wo T2DM/CVD	CHD	0.838389	7.501423e-24
wo T2DM/CVD	Is. Stroke	0.918009	2.554524e-21
wo T2DM/CVD	AF	0.930289	6.866040e-31
wo T2DM/CVD	HF	0.902909	2.643036e-23
w T2DM	CVD+AF+HF	0.915271	3.600610e-12
w T2DM	CVD	0.775940	5.796529e-05
w T2DM	CHD	0.896104	3.953026e-08
w T2DM	Is. Stroke	0.910256	4.596020e-11
w T2DM	AF	0.599110	6.832100e-06
w T2DM	HF	0.904433	1.712189e-11
w T2DM&CVD	CVD+AF+HF	0.862086	2.245521e-13
w T2DM&CVD	CVD	0.603261	7.185479e-06
w T2DM&CVD	CHD	0.814460	9.281902e-11
w T2DM&CVD	Is. Stroke	0.794963	4.388092e-15
w T2DM&CVD	AF	0.866790	6.928978e-15
w T2DM&CVD	HF	0.808064	5.725644e-11

Individuals are stratified as followed ”wo T2DM/CVD”: participants without T2DM or CVD at baseline, ”w T2DM”: participants with diabetes at baseline, ”w T2DM&CVD”: participants with T2DM at baseline and a history of CVD. The analysed outcomes include cardiovascular disease including heart failure (HF) and/or atrial fibrillation (AF) (CVD+), cardiovascular disease (CVD), coronary heart disease (CHD), HF, AF, and Ischaemic Stroke.

Supplementary Table 10: Discrimination multivariable elastic net models predicting 10-years risk of six types of CVD.

Group	Outcome	Train c-statistic	Test c-statistic	Diff c-statistic
wo T2DM/CVD	CVD+AF+HF	0.753 (0.753; 0.753)	0.750 (0.750; 0.750)	0.003
w T2DM	CVD+AF+HF	0.711 (0.710; 0.711)	0.695 (0.694; 0.696)	0.016
w T2DM&CVD	CVD+AF+HF	0.718 (0.717; 0.718)	0.677 (0.675; 0.678)	0.041
wo T2DM/CVD	CVD	0.755 (0.755; 0.755)	0.752 (0.752; 0.752)	0.003
w T2DM	CVD	0.700 (0.700; 0.701)	0.685 (0.684; 0.686)	0.015
w T2DM&CVD	CVD	0.710 (0.710; 0.711)	0.671 (0.670; 0.673)	0.039
wo T2DM/CVD	CHD	0.757 (0.757; 0.757)	0.753 (0.752; 0.753)	0.004
w T2DM	CHD	0.687 (0.687; 0.688)	0.656 (0.655; 0.657)	0.031
w T2DM&CVD	CHD	0.701 (0.700; 0.701)	0.659 (0.658; 0.660)	0.042
wo T2DM/CVD	HF	0.801 (0.801; 0.802)	0.796 (0.795; 0.797)	0.005
w T2DM	HF	0.803 (0.802; 0.804)	0.752 (0.750; 0.754)	0.051
w T2DM&CVD	HF	0.766 (0.765; 0.767)	0.746 (0.744; 0.747)	0.020
wo T2DM/CVD	AF	0.773 (0.773; 0.773)	0.766 (0.766; 0.766)	0.007
w T2DM	AF	0.763 (0.762; 0.763)	0.709 (0.707; 0.710)	0.054
w T2DM&CVD	AF	0.731 (0.731; 0.732)	0.689 (0.688; 0.691)	0.042
wo T2DM/CVD	Is. Stroke	0.765 (0.765; 0.765)	0.755 (0.754; 0.756)	0.010
w T2DM	Is. Stroke	0.768 (0.767; 0.769)	0.709 (0.706; 0.711)	0.059
w T2DM&CVD	Is. Stroke	0.810 (0.809; 0.811)	0.626 (0.624; 0.628)	0.184

Individuals are stratified as followed "wo T2DM/CVD": participants without T2DM or CVD at baseline, "w T2DM": participants with diabetes at baseline, "w T2DM&CVD": participants with T2DM at baseline and a history of CVD. The analysed outcomes include cardiovascular disease including heart failure (HF) and/or atrial fibrillation (AF) (CVD+), cardiovascular disease (CVD), coronary heart disease (CHD), HF, AF, and Ischaemic Stroke. Train discrimination (c-statistic) is based on 80% of train set of the total data used for this study, while test discrimination is calculated using remaining 20% of the total dataset. Point estimates are presented alongside 95% CI.

Supplementary Table 11: Parameters configuration for exhaustive search over parameter values for an estimator for random forest models.

Group Name	bootstrap	max_depth	min_samples_leaf	min_samples_split	n_estimators	max_samples	max_features
wo T2DM/CVD	True	3, 4, 5	50, 100	50, 70, 90, 100	50, 100	0.2, 0.5	5, 10, 15
w T2DM	True	3, 4, 5	50, 100, 150, 200	50, 70, 90, 100	50, 100, 150, 200	0.2, 0.5, 0.7, 0.99	5, 10, 15, 20, 25
w T2DM&CVD	True	3, 4, 5	50, 100, 150, 200	50, 70, 90, 100	50, 100, 150, 200	0.2, 0.5, 0.7, 0.99	5, 10, 15, 20, 25

Individuals are stratified as followed "wo T2DM/CVD": participants without T2DM or CVD at baseline, "w T2DM": participants with diabetes at baseline, "w T2DM&CVD": participants with T2DM at baseline and a history of CVD. Grid search parameters include the maximum depth of the tree (max_depth), the minimum number of samples required to split an internal node (min_samples_split), the number of trees in the forest (n_estimators), the number of samples to draw from input data to train each base estimator (max_samples), and the numbers of features to consider when looking for the best split (max_features).

Supplementary Table 12: Optimal configuration for the random forest models across three subgroups: ”wo T2DM/CVD”, ”w T2DM”, and ”w T2DM&CVD” and six CVD outcomes, identified through an exhaustive search over parameter values for an estimator.

Group Name	Outcome	bootstrap	max_depth	max_features	max_samples	min_samples_leaf	min_samples_split	n_estimators
wo T2DM/CVD	CVD+AF+HF	True	5	5	0.20	50	70	100
w T2DM	CVD+AF+HF	True	5	15	0.20	50	90	200
w T2DM&CVD	CVD+AF+HF	True	5	20	0.50	50	90	100
wo T2DM/CVD	CVD	True	5	5	0.20	50	100	100
w T2DM	CVD	True	5	20	0.20	50	100	150
w T2DM&CVD	CVD	True	5	5	0.70	50	70	150
wo T2DM/CVD	CHD	True	5	5	0.50	100	90	100
w T2DM	CHD	True	5	5	0.99	200	50	200
w T2DM&CVD	CHD	True	5	5	0.70	50	100	200
wo T2DM/CVD	HF	True	5	10	0.20	100	70	100
w T2DM	HF	True	5	20	0.50	100	50	200
w T2DM&CVD	HF	True	4	5	0.99	200	50	200
wo T2DM/CVD	AF	True	5	15	0.20	50	90	100
w T2DM	AF	True	5	10	0.50	100	50	150
w T2DM&CVD	AF	True	5	15	0.70	100	50	200
wo T2DM/CVD	Is. Stroke	True	5	10	0.20	100	50	100
w T2DM	Is. Stroke	True	5	20	0.50	150	50	200
w T2DM&CVD	Is. Stroke	True	3	5	0.99	100	70	150

Individuals are stratified as followed ”wo T2DM/CVD”: participants without T2DM or CVD at baseline, ”w T2DM”: participants with diabetes at baseline, ”w T2DM&CVD”: participants with T2DM at baseline and a history of CVD. The analysed outcomes include cardiovascular disease including heart failure (HF) and/or atrial fibrillation (AF) (CVD+), cardiovascular disease (CVD), coronary heart disease (CHD), HF, AF, and Ischaemic Stroke. Grid search parameters include the maximum depth of the tree (max_depth), the minimum number of samples required to split an internal node (min_samples_split), the number of trees in the forest (n_estimators), the number of samples to draw from input data to train each base estimator (max_samples), and the numbers of features to consider when looking for the best split (max_features).

Supplementary Table 13: Discrimination multivariable random forest models predicting 10-years risk of six types of CVD.

Group	Outcome	Train c-statistic	Test c-statistic	Diff c-statistic
wo T2DM/CVD	CVD+AF+HF	0.732 (0.732; 0.733)	0.727 (0.726; 0.727)	0.005
w T2DM	CVD+AF+HF	0.734 (0.733; 0.734)	0.684 (0.684; 0.685)	0.050
w T2DM&CVD	CVD+AF+HF	0.770 (0.769; 0.770)	0.665 (0.663; 0.666)	0.105
wo T2DM/CVD	CVD	0.738 (0.738; 0.738)	0.732 (0.732; 0.732)	0.006
w T2DM	CVD	0.729 (0.729; 0.730)	0.674 (0.673; 0.675)	0.055
w T2DM&CVD	CVD	0.784 (0.783; 0.784)	0.658 (0.657; 0.659)	0.126
wo T2DM/CVD	CHD	0.741 (0.741; 0.741)	0.732 (0.732; 0.732)	0.009
w T2DM	CHD	0.739 (0.738; 0.739)	0.631 (0.630; 0.632)	0.108
w T2DM&CVD	CHD	0.794 (0.793; 0.794)	0.652 (0.651; 0.653)	0.142
wo T2DM/CVD	HF	0.796 (0.796; 0.796)	0.782 (0.781; 0.782)	0.014
w T2DM	HF	0.844 (0.843; 0.845)	0.737 (0.735; 0.739)	0.107
w T2DM&CVD	HF	0.767 (0.766; 0.767)	0.747 (0.746; 0.748)	0.020
wo T2DM/CVD	AF	0.751 (0.751; 0.751)	0.742 (0.741; 0.742)	0.009
w T2DM	AF	0.786 (0.785; 0.786)	0.714 (0.713; 0.715)	0.072
w T2DM&CVD	AF	0.752 (0.752; 0.753)	0.679 (0.677; 0.680)	0.073
wo T2DM/CVD	Is. Stroke	0.765 (0.765; 0.766)	0.738 (0.738; 0.739)	0.027
w T2DM	Is. Stroke	0.867 (0.866; 0.868)	0.700 (0.698; 0.702)	0.167
w T2DM&CVD	Is. Stroke	0.790 (0.789; 0.791)	0.627 (0.625; 0.630)	0.163

Individuals are stratified as followed "wo T2DM/CVD": participants without T2DM or CVD at baseline, "w T2DM": participants with diabetes at baseline, "w T2DM&CVD": participants with T2DM at baseline and a history of CVD. The analysed outcomes include cardiovascular disease including heart failure (HF) and/or atrial fibrillation (AF) (CVD+), cardiovascular disease (CVD), coronary heart disease (CHD), HF, AF, and Ischaemic Stroke. Train discrimination (c-statistic) is based on 80% of train set of the total data used for this study, while test discrimination is calculated using remaining 20% of the total dataset. Point estimates are presented alongside 95% CI.

Supplementary Table 14: Comparison of feature importance ranks identified by elastic net models and random forest models based on regression coefficients and c-statistics using Spearman's correlation coefficient.

Group Name	Outcome	Spearman's rank correlation	Spearman's p-value
wo T2DM/CVD	CVD+AF+HF	0.641549	9.679838e-14
wo T2DM/CVD	CVD	0.594148	2.742226e-10
wo T2DM/CVD	CHD	0.632283	2.891801e-12
wo T2DM/CVD	Ischaemic Stroke	0.612965	2.224289e-06
wo T2DM/CVD	AF	0.636813	1.266936e-10
wo T2DM/CVD	HF	0.694711	2.040998e-09
w T2DM	CVD+AF+HF	0.608374	4.627684e-04
w T2DM	CVD	0.613534	4.013735e-03
w T2DM	CHD	0.691700	3.627009e-04
w T2DM	Ischaemic Stroke	0.546019	8.567652e-03
w T2DM	AF	0.475969	1.092148e-03
w T2DM	HF	0.410848	1.579208e-02
w T2DM&CVD	CVD+AF+HF	0.294671	7.250915e-02
w T2DM&CVD	CVD	0.434732	5.186978e-04
w T2DM&CVD	CHD	0.465390	1.214010e-04
w T2DM&CVD	Ischaemic Stroke	0.331511	1.256505e-02
w T2DM&CVD	AF	0.474278	1.144261e-03
w T2DM&CVD	HF	0.815954	4.326296e-10

Individuals are stratified as followed ”wo T2DM/CVD”: participants without T2DM or CVD at baseline, ”w T2DM”: participants with diabetes at baseline, ”w T2DM&CVD”: participants with T2DM at baseline and a history of CVD. The analysed outcomes include cardiovascular disease including heart failure (HF) and/or atrial fibrillation (AF) (CVD+), cardiovascular disease (CVD), coronary heart disease (CHD), HF, AF, and Ischaemic Stroke.

Supplementary Table 15: The number of features for the 10-years risk of cardiovascular disease unique to people with diabetes, stratified by disease type.

	CVD+AF+HF	CVD	CHD	HF	AF	Is. Stroke
Features specific to w T2DM	1	2	2	10	21	12
Features specific to w T2DM&CVD	18	29	30	34	31	75
Common features for T2DM w/wo CVD	1	0	1	7	7	10
Union of features for T2DM w/wo CVD	77	90	88	100	121	144

Participant subgroups: people without diabetes or a history of CVD at enrolment ("wo T2DM/CVD"), people with diabetes but without a history of CVD at enrolment ("w T2DM"), and people with a history of diabetes and CVD at enrolment ("w T2DM&CVD"). Rows: "Features specific to w T2DM" – the number of unique features selected for "w T2DM" group excluding features selected for other groups; "Features specific to "w T2DM&CVD" – the number of unique features selected for "w T2DM&CVD" group excluding features selected for other two groups; "Common features for T2DM w/wo CVD" – the number of common features selected for "w T2DM" and "w T2DM&CVD" excluding features identified for "wo T2DM/CVD"; "Union of features for T2DM w/wo CVD" – the number of all unique features identified for "w T2DM" and "w T2DM&CVD" excluding features selected for "wo T2DM/CVD". Outcomes: cardiovascular disease including heart failure and/or atrial fibrillation (CVD+AF+HF), cardiovascular disease (CVD), coronary heart disease (CHD), heart failure (HF), atrial fibrillation (AF), ischaemic stroke (Is. Stroke).

Supplementary Table 16: Ranks of the Qrisk3, ASCVD, and Framingham variables identified in "wo T2DM/CVD" group for CVD outcome.

Risk score variable	UK Biobank variable	Mean permuted feature importance (change in c-stat)	Std after permuted feature importance (change in c-stat)	Feature rank	Feature rank (%)	Qrisk3 variable	ASCVD variable	Framingham variable
age	age_defined_baseline	0.068279	0.002774	1.0	0.68	✓	✓	✓
sex	genetic_sex	0.024447	0.00123	2.0	1.37	✓	✓	✓
SBP	4080-0.0	0.006797	0.00058	3.0	2.05	✓	✓	✓
Family history of CVD (paternal)	20107.1	0.00326	0.000302	4.0	2.74	✓		
HDL cholesterol	30760-0.0	0.003015	0.000279	5.0	3.42	✓	✓	✓
Family history of CVD (sibling)	20111.1	0.00218	0.000284	7.0	4.79	✓		
Family history of CVD (maternal)	20110.1	0.001595	0.000229	10.0	6.85	✓		
LDL cholesterol	30780-0.0	0.00026	0.000057	38.0	26.03		✓	
DBP	4079-0.0	0.000046	0.000068	77.0	52.74	✓	✓	✓
Towsend social deprivation score	26410-0.0	not selected				✓		
Severe mental illness (moderate depression)	20123-0.0	not selected				✓		
Severe mental illness (biopolar disorder)	20122-0.0	not selected				✓		
smoking status	20116-0.0	not selected				✓	✓	✓
Severe mental illness (moderate depression)	20124-0.0	not selected				✓		
smoking status	1239-0.0	not selected				✓	✓	✓
Total cholesterol	30690-0.0	not selected				✓	✓	✓
BMI	21001-0.0	not selected				✓		
Ethnicity	21000-0.0	not selected				✓	✓	
Severe mental illness (severe depression)	20125-0.0	not selected				✓		

Feature importance was calculated using a permuted feature importance algorithm recording the change in c-statistic. The features were ranked on their relevance for CVD prediction in "wo T2DM/CVD" separately considering positive feature importance. Abbreviations: people without diabetes or a history of CVD at enrolment ("wo T2DM/CVD"), feature importance mean (Mean), feature importance standard deviation (Std), systolic blood pressure (SBP), cardiovascular disease (CVD), high-density lipoprotein cholesterol (HDL cholesterol), diastolic blood pressure (DBP).

Supplementary Table 17: Ranks of the Qrisk3, ASCVD, and Framingham variables identified in ”w T2DM” group for CVD outcome.

Risk score variable	UK Biobank variable	Mean permuted feature importance (change in c-stat)	Std after permuted feature importance (change in c-stat)	Feature rank	Feature rank (%)	Qrisk3 variable	ASCVD variable	Framingham variable
age	age_defined_baseline	0.025023	0.007535	2.0	6.9	✓	✓	✓
sex	genetic_sex	0.020663	0.003344	3.0	10.34	✓	✓	✓
DBP	4079-0.0	0.000508	0.000191	18.0	62.07	✓	✓	✓
Family history of CVD (sibling)	20111.1	0.00017	0.000166	24.0	82.76	✓		
HDL cholesterol	30760-0.0	0.000155	0.000143	25.0	86.21	✓	✓	✓
Family history of CVD (maternal)	20110.1	0.000107	0.000952	27.0	93.1	✓		
Towsend social deprivation score	26410-0.0	not selected				✓		
Severe mental illness (moderate depression)	20123-0.0	not selected				✓		
Family history of CVD (paternal)	20107.1	not selected				✓		
Severe mental illness (biopolar disorder)	20122-0.0	not selected				✓		
smoking status	20116-0.0	not selected				✓	✓	✓
Severe mental illness (moderate depression)	20124-0.0	not selected				✓		
SBP	4080-0.0	not selected				✓	✓	✓
smoking status	1239-0.0	not selected				✓	✓	✓
Total cholesterol	30690-0.0	not selected				✓	✓	✓
LDL cholesterol	30780-0.0	not selected					✓	
BMI	21001-0.0	not selected				✓		
Ethnicity	21000-0.0	not selected				✓	✓	
Severe mental illness (severe depression)	20125-0.0	not selected				✓		

Feature importance was calculated using a permuted feature importance algorithm recording the change in c-statistic. The features were ranked on their relevance for CVD prediction in ”w T2DM” separately considering positive feature importance. Abbreviations: people with diabetes but without a history of CVD at enrolment (”w T2DM”), feature importance mean (Mean), feature importance standard deviation (Std), systolic blood pressure (SBP), cardiovascular disease (CVD), high-density lipoprotein cholesterol (HDL cholesterol), diastolic blood pressure (DBP).

Supplementary Table 18: Ranks of the Qrisk3, ASCVD, and Framingham variables identified in ”w T2DM&CVD” group for CVD outcome.

Risk score variable	UK Biobank variable	Mean permuted feature importance (change in c-stat)	Std after permuted feature importance (change in c-stat)	Feature rank	Feature rank (%)	Qrisk3 variable	ASCVD variable	Framingham variable
sex	genetic_sex	0.015323	0.006561	1.0	1.23	✓	✓	✓
Family history of CVD (sibling)	20111.1	0.008713	0.003205	2.0	2.47	✓		
Family history of CVD (maternal)	20110.1	0.005137	0.001677	5.0	6.17	✓		
age	age_defined_baseline	0.004115	0.003632	8.0	9.88	✓	✓	✓
Family history of CVD (paternal)	20107.1	0.003603	0.00114	11.0	13.58	✓		
HDL cholesterol	30760-0.0	0.003107	0.002701	15.0	18.52	✓	✓	✓
DBP	4079-0.0	0.000667	0.004073	48.0	59.26	✓	✓	✓
SBP	4080-0.0	0.000616	0.0005	52.0	64.2	✓	✓	✓
Towsend social deprivation score	26410-0.0	not selected				✓		
Severe mental illness (moderate depression)	20123-0.0	not selected				✓		
Severe mental illness (biopolar disorder)	20122-0.0	not selected				✓		
smoking status	20116-0.0	not selected				✓	✓	✓
Severe mental illness (moderate depression)	20124-0.0	not selected				✓		
smoking status	1239-0.0	not selected				✓	✓	✓
Total cholesterol	30690-0.0	not selected				✓	✓	✓
LDL cholesterol	30780-0.0	not selected					✓	
BMI	21001-0.0	not selected				✓		
Ethnicity	21000-0.0	not selected				✓	✓	
Severe mental illness (severe depression)	20125-0.0	not selected				✓		

Feature importance was calculated using a permuted feature importance algorithm recording the change in c-statistic. The features were ranked on their relevance for CVD prediction in ”w T2DM&CVD” separately considering positive feature importance. Abbreviations: people with diabetes and a history of CVD at enrolment (”w T2DM&CVD”), feature importance mean (Mean), feature importance standard deviation (Std), systolic blood pressure (SBP), cardiovascular disease (CVD), high-density lipoprotein cholesterol (HDL cholesterol), diastolic blood pressure (DBP).

1.4 Supplementary Figures

Supplementary Figure 1: Overview of the study design pipeline.

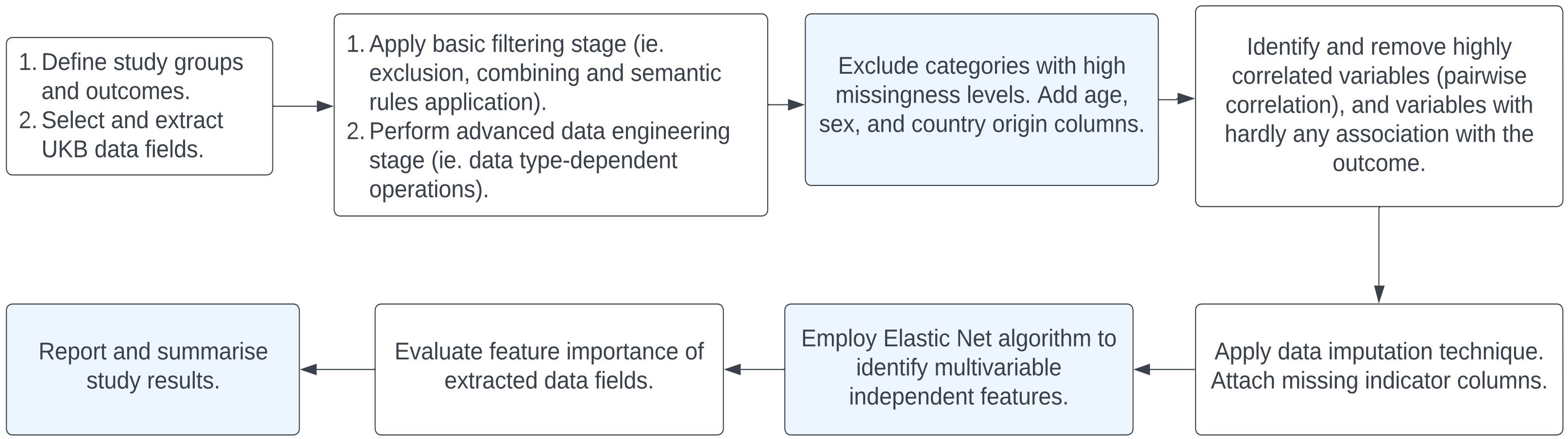

Supplementary Figure 2: Flow diagram of the initial filtering and standardization step.

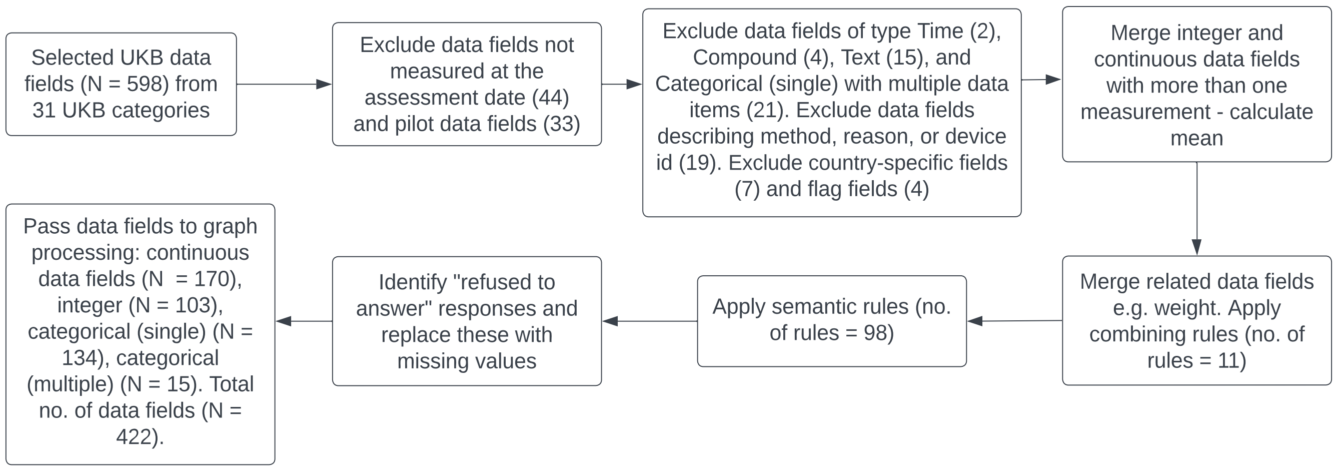

Supplementary Figure 3: Flow diagram of data type-specific transformation step.

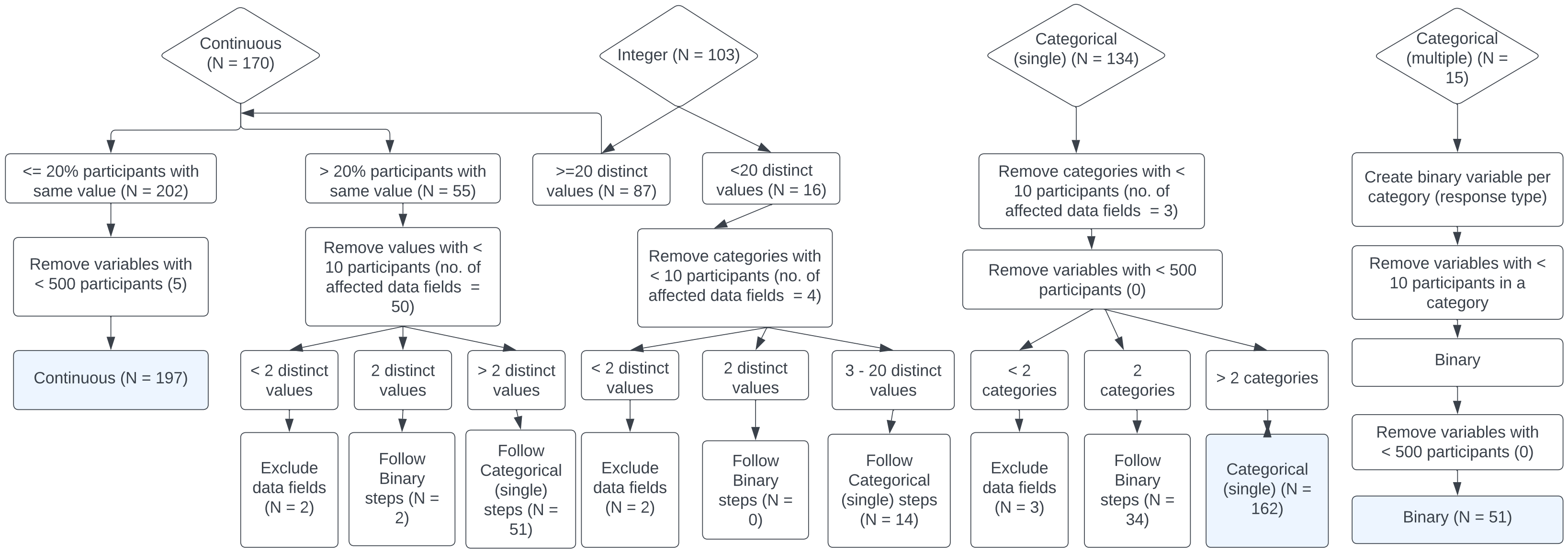

Supplementary Figure 4: Overview of features available at the different stages of our feature selection algorithm.

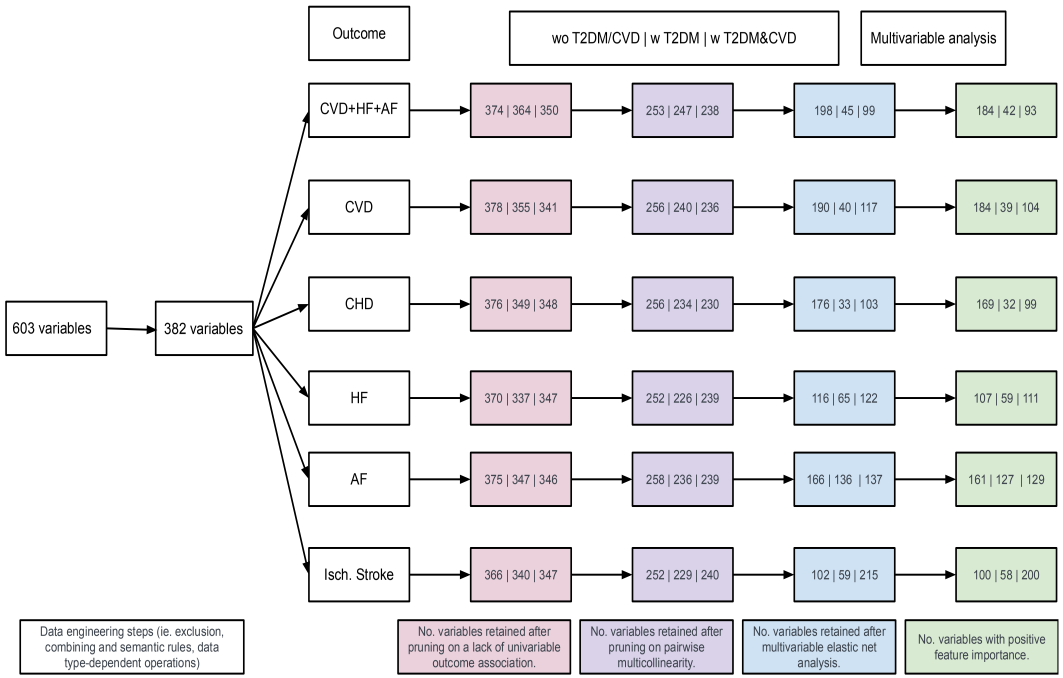

Variables were removed based on an insufficient univariable association with the outcome (Spearman's p-value greater than 0.8), because of a high pairwise correlation (Spearman's correlation above 0.70), through elastic net based penalization, or because of a negative feature importance in the testing data (representing independent replication). Abbreviations: type 2 diabetes (T2DM); cardiovascular disease (CVD); coronary heart disease (CHD); atrial fibrillation (AF); heart failure (HF); ischemic stroke (Isch. Stroke).

Supplementary Figure 5: Top 40 most predictive features for the 10-years risk of cardiovascular disease identified by random forest models.

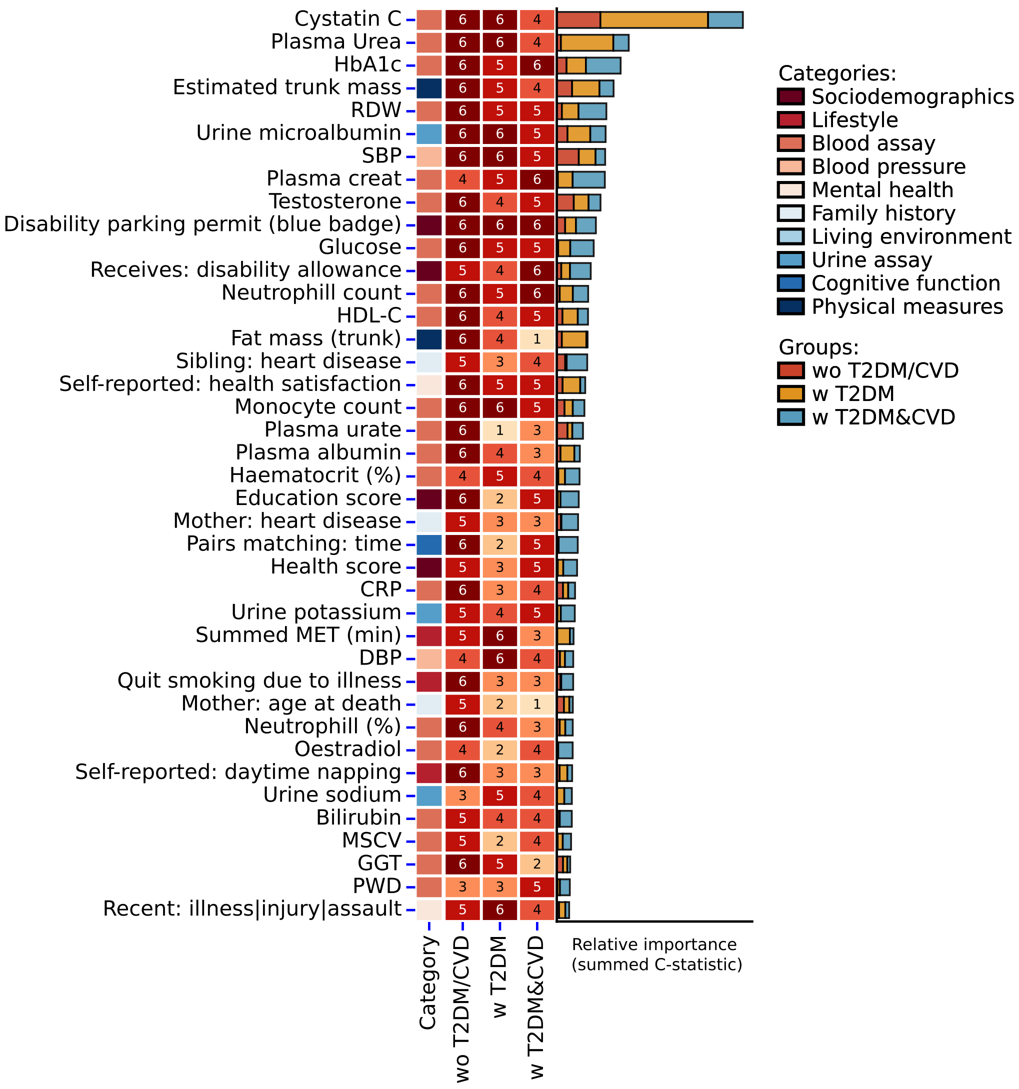

The y-axis presents the top 40 features (excluding age and sex) identified by random forest models and based on the summed feature importance aggregated across the six types of CVD considered (CVD+AF+HF, CVD, CHD, Isch. Stroke, HF, AF) stratified by participants subgroup: people without diabetes or a history of CVD at enrolment ("wo T2DM/CVD"), people with diabetes but without a history of CVD at enrolment ("w T2DM"), and people with a history of diabetes and CVD at enrolment ("w T2DM&CVD"). The heatmap (mid panel) represents the number of CVD outcomes the features was identified for (at most 6), while the stacked bar chart (right panel) encodes the summed feature importance, stratified by participant subgroup. Feature importance was calculated using a permuted feature importance algorithm recording the change in c-statistic. The algorithm was applied to the hold-out test data and hence represent an unbiased estimates of the feature importance as well as reflecting features which were independently replicated.

Supplementary Figure 6: The contribution of the top features to the prediction of six facets of CVD for "wo T2DM/CVD" group.

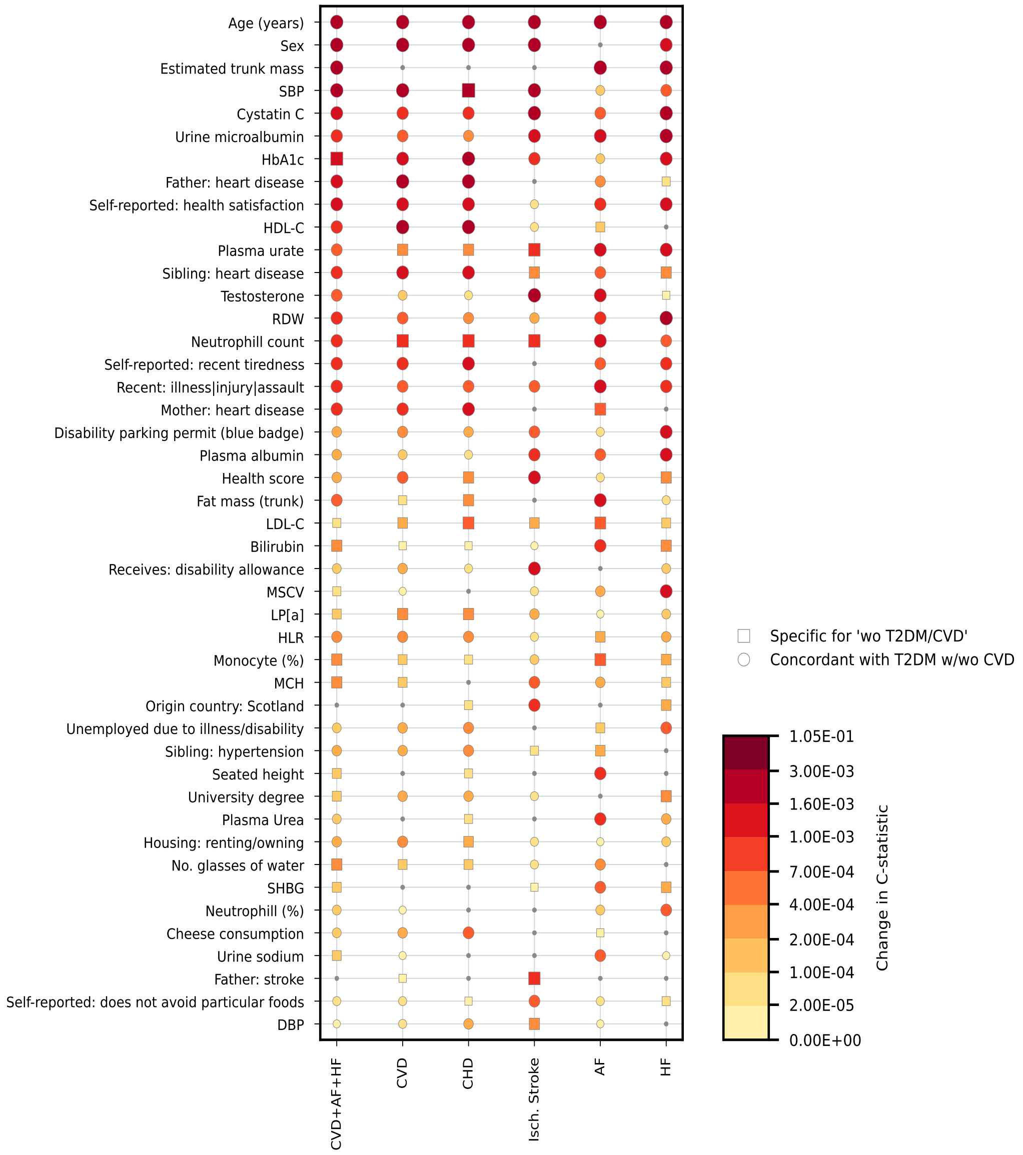

The y-axis presents the union of the top 20 features based on the c-statistic change for each of the six CVD outcomes, features in violet originate from the NMR UKB category. Plot markers (e.g. triangle, square) highlight the differences in identified features between groups. The permuted feature importance reflects the c-statistic change based on the test data; iteratively the values of each variable were randomly assigned to an individual after which the c-statistic was re-estimated with these permuted data and the difference in performance was used as an estimate of a variable contribution to the model's predictive potential. Abbreviations: glycated haemoglobin (HbA1c), high-density lipoprotein cholesterol (HDL-C), red blood cell distribution width (RDW), systolic blood pressure (SBP), sex hormone-binding globulin (SHBG), mean corpuscular haemoglobin (MCH), lipoprotein A (LP[a]), low-density lipoprotein cholesterol (LDL-C), platelet crit (PCT), mean platelet (thrombocyte) volume (MPV), mean sphered cell volume (MSCV).

Supplementary Figure 7: The contribution of the top 60 features to the prediction of six facets of CVD for "w T2DM" group.

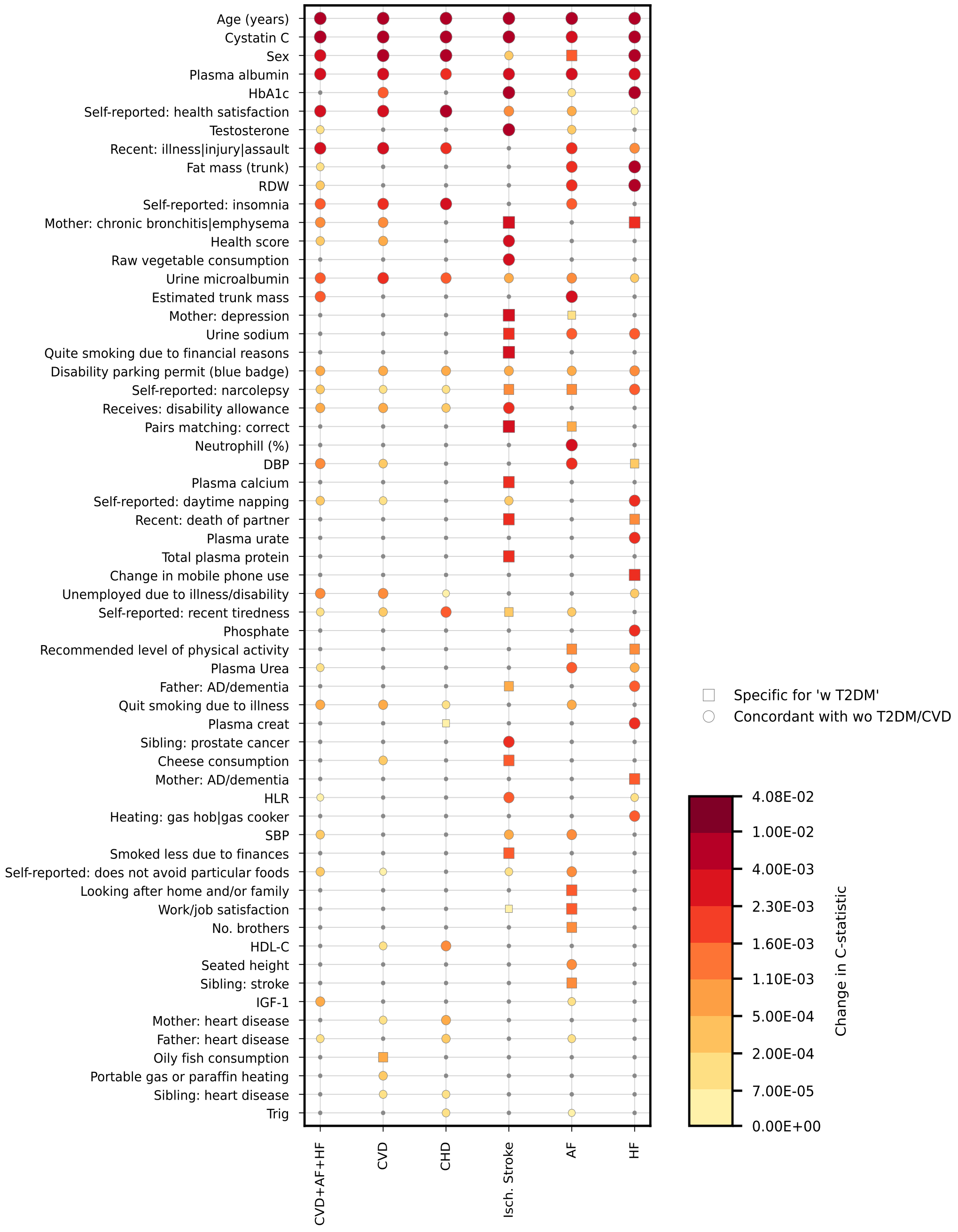

The y-axis presents the union of the top 20 features (limited to maximum 60 features) based on the c-statistic change for each of the six CVD outcomes. Plot markers (e.g. triangle, square) highlight the differences in identified features between groups. The permuted feature importance reflects the c-statistic change based on the test data; iteratively the values of each variable were randomly assigned to an individual after which the c-statistic was re-estimated with these permuted data and the difference in performance was used as an estimate of a variable contribution to the model's predictive potential. Abbreviations: glycated haemoglobin (HbA1c), systolic blood pressure (SBP), red blood cell distribution width (RDW), high light scatter reticulocyte count (HLR), sex hormone-binding globulin (SHBG), Qualifications (O levels / General Certificate of Secondary Education or equivalent) (O-levels/GCSEs), high-density lipoprotein cholesterol (HDL-C), diastolic blood pressure (DBP), lipoprotein A (LP[a]).

Supplementary Figure 8: The contribution of the top 60 features to the prediction of six facets of CVD for ”w T2DM&CVD” group.

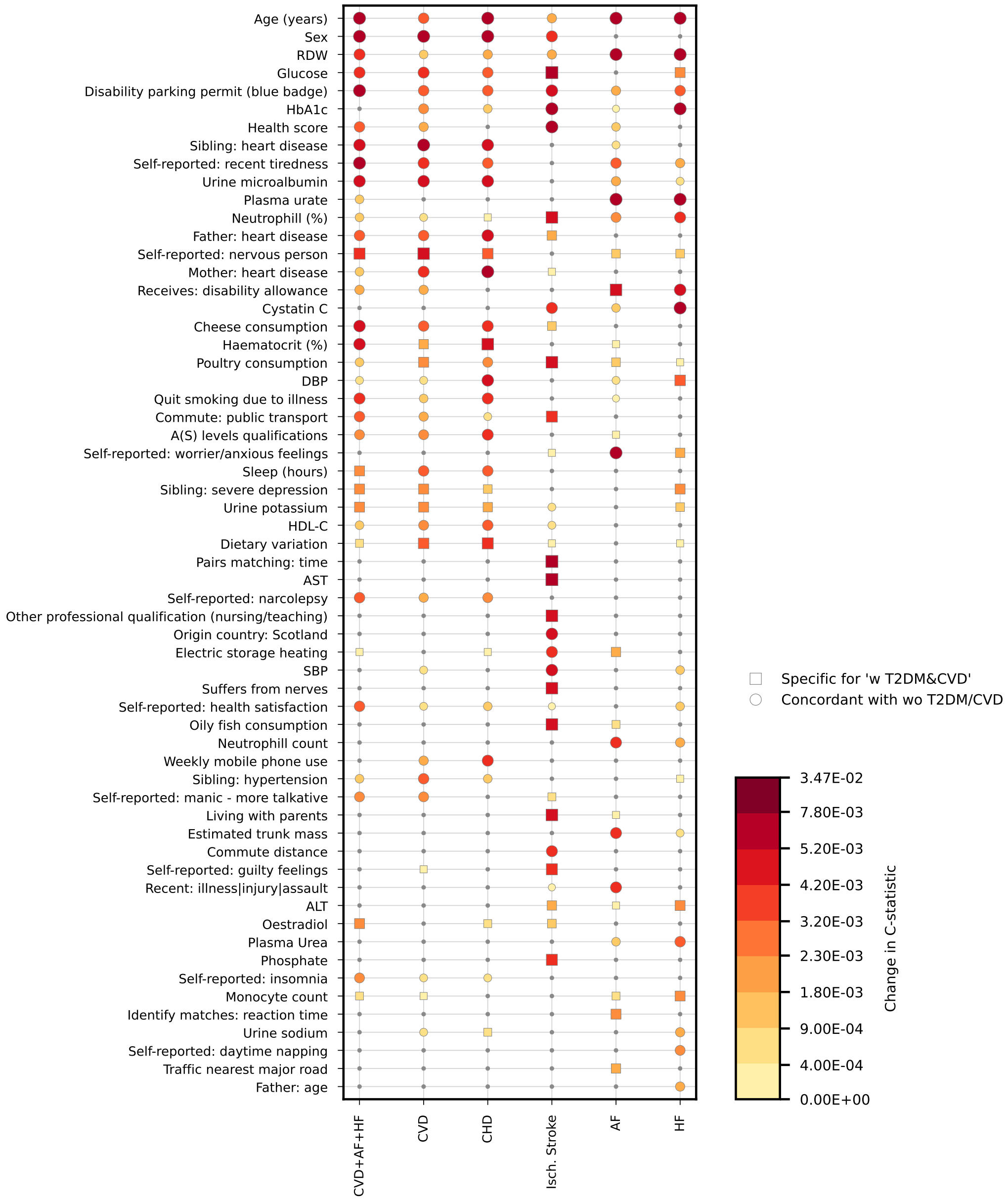

The y-axis presents the union of the top 20 features (limited to a maximum of 60 features) based on the c-statistic change for each of the six CVD outcomes. Plot markers (e.g. triangle, square) highlight the differences in identified features between groups. The permuted feature importance reflects the c-statistic change based on the test data; iteratively the values of each variable were randomly assigned to an individual after which the c-statistic was re-estimated with these permuted data and the difference in performance was used as an estimate of a variable contribution to the model’s predictive potential. Abbreviations: red blood cell distribution width (RDW), alanine aminotransferase (ALT), low-density lipoprotein cholesterol (LDL-C), gamma glutamyltransferase (GGT), systolic blood pressure (SBP), qualifications (CSEs or equivalent) (CSE-equivalent), glycated haemoglobin (HbA1c), c-reactive protein (CRP), platelet crit (PCT), qualifications (NVQ or HND or HNC or equivalent) (NVQ/HND/HNC-equivalent).

Supplementary Figure 9: Results of Wilcoxon tests on the difference in ranked feature importance identified by elastic net models between ”wo T2DM/CVD” and ”w T2DM”, and between ”wo T2DM/CVD” and ”w T2DM&CVD.”

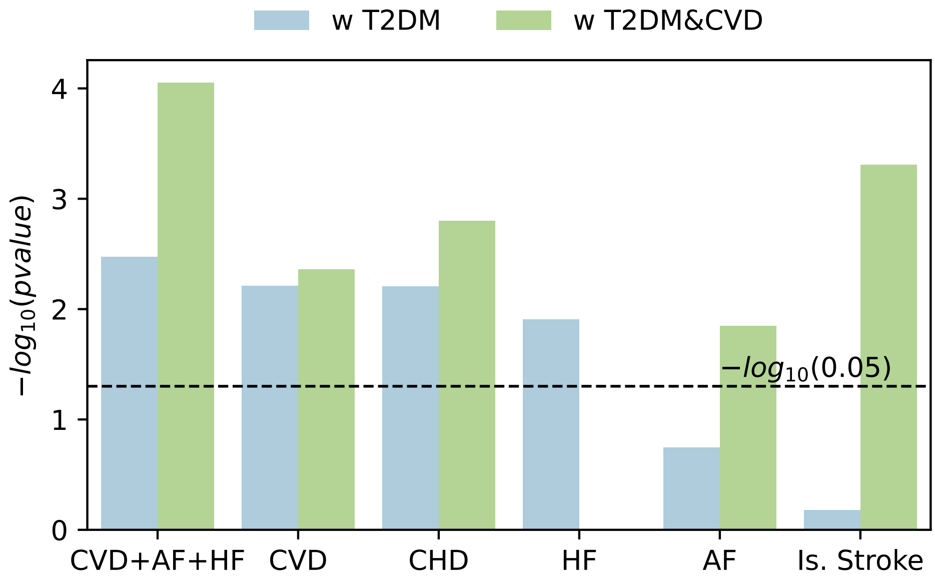

Results are stratified by type of CVD and participant subgroup. The Wilcoxon test is conducted on the difference in feature ranks identified by the elastic net model in people without diabetes or a history of CVD at enrolment (”wo T2DM/CVD”, n = 91829) compared to feature ranks identified in two other subgroups: people with diabetes but without a history of CVD at enrolment (”w T2DM”, n = 2922), and people with a history of diabetes and CVD at enrolment (”w T2DM&CVD”, n = 887).

Supplementary References

1. *UKB Data-Field 5674* https://biobank.ctsu.ox.ac.uk/crystal/field.cgi?id=5674 (2022).
2. *Accessing UK Biobank Data* https://biobank.ctsu.ox.ac.uk/~bbdatan/Accessing_UKB_data_v2.3.pdf (2022).
3. *UKB Data-Field 5076* https://biobank.ndph.ox.ac.uk/ukb/field.cgi?id=5076 (2022).
4. *UKB Data Field Value Types* https://biobank.ndph.ox.ac.uk/showcase/help.cgi?cd=value_type (2022).
5. C., K. H. & Dale, A. E. Heart Failure in Type 2 Diabetes Mellitus. *Circulation Research* **124,** 121–141 (Jan. 2019).
6. Seyed Ahmadi, S., Svensson, A.-M., Pivodic, A., Rosengren, A. & Lind, M. Risk of atrial fibrillation in persons with type 2 diabetes and the excess risk in relation to glycaemic control and renal function: a Swedish cohort study. **19,** 9. ISSN: 1475-2840 (Jan. 2020).
7. Millard, L. A., Davies, N. M., Gaunt, T. R., Davey Smith, G. & Tilling, K. Software Application Profile: PHESANT: a tool for performing automated phenome scans in UK Biobank. *International Journal of Epidemiology* **47,** 29–35. ISSN: 0300-5771 (Feb. 2018).
8. *UKB Data-Field 6158 - Why reduced smoking* https://biobank.ctsu.ox.ac.uk/crystal/field.cgi?id=6158 (2022).

9. Buuren, S. v. & Groothuis-Oudshoorn, K. mice: Multivariate Imputation by Chained Equations in R. en. *Journal of Statistical Software* **45,** 1–67. ISSN: 1548-7660 (Dec. 2011).

10. Zou, H. & Hastie, T. Regularization and Variable Selection via the Elastic Net. *Journal of the Royal Statistical Society. Series B (Statistical Methodology)* **67,** 301–320. ISSN: 1369-7412 (2005).

11. Arnett, D. K. *et al.* 2019 ACC/AHA Guideline on the Primary Prevention of Cardiovascular Disease: A Report of the American College of Cardiology/American Heart Association Task Force on Clinical Practice Guidelines. *Circulation* **140,** e596–e646 (2019).

12. Development and validation of QRISK3 risk prediction algorithms to estimate future risk of cardiovascular disease: prospective cohort study. en. **357,** j2099. ISSN: 1756-1833 (May 2017).

13. Prediction of Coronary Heart Disease Using Risk Factor Categories. **97,** 1837–1847 (May 1998).

14. Cook, N. R. Quantifying the added value of new biomarkers: how and how not. *Diagnostic and Prognostic Research* **2,** 14. ISSN: 2397-7523 (2018).
