# Supplementary material for: Identifying and ranking non-traditional risk factors for cardiovascular disease prediction in people with type 2 diabetes
Source: Commun Med (Lond). 2025 Mar 14;5:77. doi: 10.1038/s43856-025-00785-y (PMC11906859; doi:10.1038/s43856-025-00785-y)
Supplement: Supplementary file 2 — Description of Additional Supplementary Files [file 43856_2025_785_MOESM2_ESM.pdf]

Description of Additional Supplementary Files

File name: Supplementary Data 1

File description– Semantic rules to process and standardize the UK Biobank data fields.

File name: Supplementary Data 2

File description– Feature importance of variables identified by the Elastic Net model for individuals without type 2 diabetes and a history of cardiovascular disease (“wo T2DM/CVD”), individuals with type 2 diabetes but not history of cardiovascular disease (“w T2DM”), and individuals with type 2 diabetes and a history of cardiovascular disease (“w T2DM&CVD”).

File name: Supplementary Data 3

File description– Elastic Net model coefficients for individuals without type 2 diabetes and a history of cardiovascular disease (“wo T2DM/CVD”), individuals with type 2 diabetes but not history of cardiovascular disease (“w T2DM”), and individuals with type 2 diabetes and a history of cardiovascular disease (“w T2DM&CVD”).

File name: Supplementary Data 4

File description– Feature importance of variables identified by the Random Forest model for individuals without type 2 diabetes and a history of cardiovascular disease (“wo T2DM/CVD”), individuals with type 2 diabetes but not history of cardiovascular disease (“w T2DM”), and individuals with type 2 diabetes and a history of cardiovascular disease (“w T2DM&CVD”).

File name: Supplementary Data 5

File description– Common features selected for all three participant groups, stratifying by CVD outcome type.

File name: Supplementary Data 6

File description– Features uniquely selected for individuals with type 2 diabetes but without a history of cardiovascular disease (“w T2DM”).

File name: Supplementary Data 7

File description– Features uniquely selected for individuals with type 2 diabetes and a history of cardiovascular disease (“w T2DM&CVD”).

File name: Supplementary Data 8

File description– Common features selected for diabetes groups; individuals with type 2 diabetes but without a history of cardiovascular disease (“w T2DM”) and individuals with type 2 diabetes and cardiovascular disease (“w T2DM&CVD”).

File name: Supplementary Software 1

File description– https://gitlab.com/cvd_in_t2dm/novel_features_cvd_prediction
