# Supplementary material for: Identifying and ranking non-traditional risk factors for cardiovascular disease prediction in people with type 2 diabetes
Source: Commun Med (Lond). 2025 Mar 14;5:77. doi: 10.1038/s43856-025-00785-y (PMC11906859; doi:10.1038/s43856-025-00785-y)
Supplement: Supplementary file 12 — Supplementary Software Information [file 43856_2025_785_MOESM12_ESM.docx]

*Identifying and ranking non-traditional risk factors for cardiovascular disease prediction in people with type 2 diabetes*

Katarzyna Dziopa

Link to DOI: [10.5281/zenodo.14779981](https://doi.org/10.5281/zenodo.14779981)
